# Extreme Heat, Social Factors, and Mortality Among California Veterans With Cardiometabolic Disease

**DOI:** 10.1001/jamanetworkopen.2025.45524

**Published:** 2025-11-25

**Authors:** Evan Michael Shannon, Lillian Chen, Anita Yuan, Aarthi Chary, Sonya Gabrielian, David P. Eisenman, Donna L. Washington

**Affiliations:** 1 Veterans Affairs Health Systems Research Center for the Study of Healthcare Innovation, Implementation & Policy, Veterans Affairs Greater Los Angeles Healthcare System, Los Angeles, California; 2 Division of General Internal Medicine and Health Services Research, UCLA David Geffen School of Medicine, Los Angeles, California; 3 Department of Medicine, Stanford University School of Medicine, Stanford, California; 4 Veterans Affairs Palo Alto Healthcare System, Palo Alto, California; 5 Department of Psychiatry, UCLA David Geffen School of Medicine, Los Angeles, California

## Abstract

**Importance:**

There is limited research investigating associations between extreme heat events (EHEs) and adverse outcomes for veterans and high-risk veteran subpopulations.

**Objectives:**

To investigate the association between EHEs and mortality among California veterans with common cardiometabolic diseases and to examine how this risk differs by race and ethnicity, neighborhood health-related social risk factors, and history of homelessness.

**Design, Setting, and Participants:**

This case-crossover study used administrative data from the Veterans Health Administration (VA) Corporate Data Warehouse to identify veterans with a California address from October 1, 2015, to September 30, 2021, and a diagnosis of a cardiometabolic condition of hypertension, diabetes, ischemic heart disease, congestive heart failure, chronic kidney disease, stroke, or peripheral arterial disease. Data were analyzed from October 2023 to December 2024.

**Exposure:**

Date- and address-specific EHEs as defined by daily maximum temperatures greater than the 90th, 95th, and 97.5th percentiles of 2006 to 2020 historical normal temperatures derived from National Center for Environmental Information meteorological data. Patient residential addresses were linked with the nearest weather station. The associations of same-day to 4-day EHEs with mortality were assessed.

**Main Outcomes and Measures:**

The main outcome was individual all-cause mortality during warm months (April-October). A time-stratified case-crossover design with conditional logistic regression was used to investigate associations between EHEs and mortality. Planned exploratory subgroup analyses were performed by race and ethnicity, Area Deprivation Index (ADI) for patient address, and homeless status.

**Results:**

A total of 13 556 veterans (median age, 78 years [IQR, 71-87 years]; 13 265 men [97.9%]) experienced mortality during the study period. Extreme heat events were significantly associated with mortality at all percentile thresholds (eg, at 95th percentile odds ratio [OR] range, 1.10 [95% CI, 1.04-1.17] to 1.14 [95% CI, 1.08-1.20]; *P* = .005). Effect estimates were greater for veterans in high vs lower ADI neighborhoods (eg, 3-day EHEs at 95th percentile: OR, 1.44 [95% CI, 1.15-1.80] vs OR, 1.12 [95% CI, 1.06-1.19]), and veterans who experienced homelessness compared with those who did not (eg, 3-day EHEs at 95th percentile: OR, 1.25 [95% CI, 1.09-1.45] vs OR, 1.12 [95% CI, 1.05-1.19]) across EHE definitions, although there was no statistically significant effect modification.

**Conclusions and Relevance:**

In this study of California veterans with common cardiometabolic diseases, EHEs were significantly associated with mortality. These results suggest that the VA should develop EHE risk-mitigating interventions to protect veterans, particularly those at greatest risk, from heat-related mortality.

## Key Points

**Question:**

Are extreme heat events (EHEs) associated with increased risk of mortality among California veterans with cardiometabolic disease?

**Findings:**

In this time-stratified case-crossover study of 13 556 US veterans, EHEs were significantly associated with increased risk of mortality for same-day EHEs at the 95th percentile of historical mean temperatures. Veterans residing in lower socioeconomic status (SES) neighborhoods and veterans experiencing homelessness had increased risk of mortality compared with those living in higher SES neighborhoods and housed veterans, respectively, although these differences were not statistically significant.

**Meaning:**

This study suggests that the Veterans Health Administration should consider the increasing threat of EHEs for veteran communities at highest risk.

## Teaser

This case-control study with a case-crossover design investigates the association between extreme heat events and mortality among California veterans with cardiometabolic disease and examines how this risk differs by race and ethnicity, neighborhood health-related social risk factors, and history of homelessness.

## Introduction

The Intergovernmental Panel on Climate Change projects that extreme heat events (EHEs), or periods in which temperatures are significantly higher than typical, will become increasingly common as global mean temperatures increase.^1^ Among the numerous ramifications of increasing global mean temperatures, extreme heat is the most deadly in North America.^2,3,4,5,6,7,8^ For example, approximately 1000 excess deaths were attributed to the US and Canada Pacific Northwest June-July 2021 heat wave.^9^ This risk is heightened among people with underlying cardiometabolic disease, who are less physiologically capable of adapting to the increased cardiac workload resulting from extreme heat exposure.^10^ In addition, many classes of medications used to treat cardiometabolic disorders (eg, antihypertensives, diuretics) can increase risk from heat exposure, via impaired thermoregulation, volume depletion, decreased blood pressure, or reduced cardiac output.^11^

Veterans have risk factors that place them at high risk of adverse outcomes associated with EHEs. Veterans who seek care through the Veterans Health Administration (VA) are generally older and have higher rates of cardiometabolic disease compared with nonveterans,^12^ with advanced age^2,13^ and cardiometabolic comorbidity^14,15^ recognized as risk factors. Another known risk factor that affects many veterans is homelessness.^16,17,18^ In 2024, 5.3% of US adults experiencing homelessness were veterans, 60% of whom were unsheltered.^19^ Elevated risk of heat-related illness among individuals experiencing homelessness could be due to prolonged outdoor exposure during periods of extreme heat,^17,20,21^ transportation barriers preventing access to health care facilities and cooling centers,^22^ and/or greater susceptibility to heat-related illness, due to higher prevalence of mental health disorders and receipt of antipsychotic or antidepressant medications that interfere with thermoregulation.^21^

Individuals residing in economically underserved areas also experience a disproportionate burden of heat-related morbidity and mortality.^5,23,24,25,26,27,28,29,30^ These communities tend to be in urban centers, in which heat is modulated by factors in the built environment, such as paved surfaces, thermal properties of buildings, and percentage of canopy cover.^24,31,32,33^ Generations of systemic racism in the forms of redlining^34^ and disproportionate incarceration^35^ have forced racial and ethnic minoritized communities to live in urban centers that are imbalanced toward factors that increase urban heat, contributing to “thermal inequity”^32^ and increased risk from EHE-associated morbidity among Black populations.^2,5,23,28,36^

The VA is an integrated, single-payer health care system^37^ that provides comprehensive health care to all eligible beneficiaries, which may mitigate risk from adverse outcomes due to extreme heat and disparities in these outcomes. Furthermore, as a learning health system that is dedicated to integrating research, clinical practice, and quality improvement to improve health care for veterans,^38^ the VA is an ideal environment for developing evidence-based practices to protect veterans from EHEs. Globally and nationally, public health and health care systems have implemented heat action plans to combat adverse outcomes during EHEs.^39^ To date, there is limited research on the effect of EHEs on veterans, including veterans with elevated risks due to cardiometabolic disease. Such research would provide foundational data for the VA to strengthen comprehensive heat action planning and implement evidence-based targeted interventions.

The primary aim of this study was to investigate the association between EHEs and mortality among veterans with common cardiometabolic diseases. We performed our study among veterans with cardiometabolic diseases because those disorders are common among veterans (eg, an estimated 70%-80% of VA users have hypertension^40^) and because extreme heat causes morbidity and mortality via mechanisms that cause end-organ ischemia, placing those with cardiometabolic disease at greatest risk. We also sought to explore if the association differed across key veteran subpopulations, with attention to race and ethnicity, neighborhood health-related social risk factors, and those with homeless experiences. We focused our study on California veterans because EHEs are projected to disproportionately increase in the Western US^41^ and one-third of veterans experiencing homelessness reside in California.^42^ Our primary hypothesis was that EHEs are associated with increased mortality overall.

## Methods

This study followed the Strengthening the Reporting of Observational Studies in Epidemiology (STROBE) reporting guideline for case-control studies.^43^ This study was approved by the VA Greater Los Angeles Healthcare System institutional review board. Participant consent was waived for this secondary data analysis per the VA Greater Los Angeles Healthcare System institutional review board given the patient volume and impracticability of obtaining patient consent. Patient records were deidentified during the analysis phase.

### Cohort Definition and Outcome

We used administrative and electronic health record data from the VA Corporate Data Warehouse (CDW). Our study period was from October 1, 2015, to September 30, 2021 (fiscal years 2016-2021). We identified veterans with a California residential address during the study period using the Planning Systems Support File,^44^ which includes patient residential address and is updated quarterly.

We identified veterans who had a cardiometabolic disease diagnosis: hypertension, diabetes, ischemic heart disease, congestive heart failure, chronic kidney disease (stage 3 or greater), prior stroke, or peripheral arterial disease. These diagnoses were identified using *International Statistical Classification of Diseases and Related Health Problems, Tenth Revision* (*ICD-10*) codes (eTable 1 in Supplement 1) for at least 1 inpatient encounter or at least 2 ambulatory encounters within the VA (ie, not including community care) during the study period. We excluded patients without a documented California residential address after a cardiometabolic disease diagnosis. We also calculated the Gagne comorbidity score^45^ and the Care Assessment Needs score for 90-day mortality,^46^ which is a commonly used tool that estimates patient percentile risk of mortality or hospitalization, at time of mortality. We identified whether a patient died of any cause during the study period using the CDW’s VA Vital Status Master File.

### Primary Exposure Variable: EHEs

We defined EHEs using daily maximum temperatures, consistent with prior published definitions.^7,47,48^ Using data from the National Center for Environmental Information (NCEI) through the National Oceanic Atmospheric Agency, we identified zip code–specific calendar day maximum temperatures and standard deviations of the temperature distribution based on 2006 to 2020 historical normal temperatures. We then linked patient address zip codes by fiscal year quarter with these meteorological data. If the NCEI weather station associated with a zip code was missing historical normal temperature data, we linked patient address zip code to the nearest zip code with complete meteorological data within 50 miles. We considered a day to be an extreme heat day if the maximum daily temperature exceeded 1 of the 3 definitions of “extreme”—the 90th, 95th, or 97.5th percentile thresholds. We report findings using these 3 thresholds as each has been used in prior studies^17,49,50^ as a cutoff to define an EHE and to explore variability in effect estimates by threshold. We then assessed whether a day was a singular extreme heat day or among 1 of up to 4 consecutive extreme heat days (ie, a 2-, 3-, or 4-day heat wave). We limited the analysis to the warmer months of the year (April-October) to ensure an EHE represents a high actual temperature, as has been performed in similar research.^3,23,25,32,47,51^

### Subpopulations of Interest

Patient race and ethnicity were obtained from the CDW and categorized as American Indian or Alaska Native, Asian, Black, Hispanic, Native Hawaiian or Other Pacific Islander, non-Hispanic White, and multiracial. In the CDW, race and ethnicity are typically self-reported. Race and ethnicity data were collected so that these analyses could be conducted by race and ethnicity subgroups. To estimate neighborhood health-related social risk factors, we linked patient address with their census block group to determine the patient residential Area Deprivation Index (ADI)^52,53^ at the time of death. The ADI is a validated index derived from measures of income, education, employment, and housing quality from the American Community Survey^52^ that compares neighborhoods (ie, census block groups) across the US and within a given state to develop an ordinal score, with higher values indicating greater deprivation and lower socioeconomic status. We used the national ADI, then categorized neighborhoods as highest quartile ADI (ie, ≥75) or lower ADI (ie, <75).

We used the CDW to identify veterans experiencing homelessness, which we defined as veteran patients experiencing homelessness at any point during the study period. Veterans may vacillate in and out of homelessness and between being sheltered and unsheltered.^54^ A patient was defined as experiencing homelessness if they had a VA encounter with an associated *International Classification of Diseases, Ninth Revision* (*ICD-9*), or *ICD-10* code for homelessness or a VA encounter with an inpatient or outpatient VA homeless program (identified via stop codes in the CDW), in keeping with prior studies.^55^ Addresses for patients experiencing homelessness may be the last residential address or address of a transitional or permanent supportive housing unit where they reside.

### Statistical Analysis

Statistical analysis was performed from October 2023 to December 2024. To evaluate if EHEs were associated with increased risk of mortality in patients with cardiometabolic disease, we used a time-stratified case-crossover study design, which is typical for this type of research investigating the associations of a time-limited exposure.^2,3,7,17,23,51^ In a case-crossover study design, the study cohort is limited to those who experienced the case-defining event—in this study, mortality. An individual serves as their own control, which removes the need to further adjust for time-invariant covariates such as individual sex and race and ethnicity.^56^ In this design, each “case” is defined as an adverse outcome (mortality), and the matched control is the same individual on the same day of the week for the other weeks in the same month. After this matching, the association between EHE and mortality can be assessed. If experiencing an event (ie, mortality) is not associated with the distribution of future exposure (ie, EHEs) in the overall study population, selecting postevent control times is acceptable.^57,58^ We excluded patients with missing linked complete temperature data.

To calculate the association between EHE and mortality, we performed conditional logistic regression models using matched cases and controls. We used stratum indicators based on the combination of California climate zone (16 distinct geographic regions with similar climate profiles,^59^ to account for California’s climate diversity) and year, month, and day of the week to control for day-of-week–related changes in underlying risk, which could confound the association between exposure and outcome,^60^ as is consistent with other similar studies.^7,61^ We considered the association between same-day EHEs on the day of a patient’s death, through 4-day EHEs lasting from 3 days prior to a patient’s death to the day of the mortality event, in keeping with similar studies.^62,63^

First, we performed regression models for the cohort overall. We then performed models separately by race and ethnicity, ADI, and homelessness status (excluding patients with missing subgroup variables from the respective subgroup analysis) and compared effect estimates between subgroup categories. For subgroup analyses, we also performed models including an interaction term for EHE × subgroup category to assess whether there was evidence of significant effect modification. Effect estimates are presented as unadjusted odds ratios (ORs) with 95% CIs. Results were considered statistically significant at a 2-sided α level of less than .05. For the exploratory analyses by subgroups of interest, we did not anticipate having adequate sample size to detect differences within categories and therefore present effect estimates for subgroup categories compared with the null and *P* value for interaction. All analyses were performed using R studio, version 4.4.1 (R Project for Statistical Computing).

With our dataset, we were able to use individual-level data to assess exposure rather than aggregate data by a geographic region, which typically relies on conditional Poisson or quasi-Poisson regression.^7,16,60,64,65^ One benefit of conditional Poisson modeling is that it can account for population shifts during the study period and allows for adjustments for overdispersion and autocorrelation,^7,60^ although a limitation of this design is that it does not account for the ecologic fallacy.^66^ We performed sensitivity analyses using conditional Poisson and quasi-Poisson regression aggregated by California climate zones to evaluate associations between EHEs and mortality. We also performed an analysis using only zip codes for which there were associated normal temperatures, without using nearest weather station if normal temperatures were not available.

## Results

Among the 277 876 veterans with a diagnosis of 1 or more cardiometabolic conditions who resided in California between fiscal years 2015 and 2021, 13 556 died (eFigure 1 in Supplement 1; distribution of sample across California by county in eFigure 2 in Supplement 1). Among those who died, the median age was 78 years (IQR, 71-87 years), 13 265 (97.9%) were male, and 291 (2.1%) were female (Table 1). By race and ethnicity, 1276 (9.4%) were Hispanic, 92 (0.7%) were non-Hispanic American Indian or Alaska Native, 389 (2.9%) were non-Hispanic Asian, 1687 (12.4%) were non-Hispanic Black, 139 (1.0%) were non-Hispanic Native Hawaiian or Other Pacific Islander, 8332 (61.5%) were non-Hispanic White, and 93 (0.7%) were non-Hispanic multiracial; 1548 (1.4%) had missing race and ethnicity. By cardiometabolic diagnosis, 11 847 (87.4%) had hypertension, 6338 (46.8%) had diabetes, and 4788 (35.3%) had ischemic heart disease; patients had a median of 2 (IQR, 1-3) cardiometabolic conditions. Overall, 2017 veterans (14.9%) experienced homelessness, and 1038 (7.7%) resided in a highest quartile ADI neighborhood.

**Table 1.** Characteristics of Cohort of California Veterans With Cardiometabolic Disease by Mortality Status Between Fiscal Years 2016 and 2021

Characteristic	Veterans, No. (%)	
	Mortality (n = 13 556)	No mortality (n = 264 320)
Age, median (IQR), y^a^	78 (71 to 87)	67 (58 to 73)
Sex		
Female	291 (2.1)	12 769 (4.8)
Male	13 265 (97.9)	251 551 (95.2)
Race and ethnicity		
Hispanic	1276 (9.4)	33 867 (12.8)
Non-Hispanic Asian	389 (2.9)	15 328 (5.8)
Non-Hispanic American Indian or Alaska Native	92 (0.7)	2018 (0.8)
Non-Hispanic Black	1687 (12.4)	40 212 (15.2)
Non-Hispanic Native Hawaiian or Other Pacific Islander	139 (1.0)	4341 (1.6)
Non-Hispanic White	8332 (61.5)	141 075 (53.4)
Non-Hispanic multiracial	93 (0.7)	2340 (0.9)
Missing	1548 (11.4)	25 149 (9.5)
History of homelessness	2017 (14.9)	35 229 (13.3)
National ADI, median (IQR)^a^^,^^b^	22 (11 to 39)	20 (10 to 35)
National ADI category (high, ≥75; low, <75)^a^		
High	1038 (7.7)	15 871 (6.0)
Low	12 518 (92.3)	248 449 (94.0)
Gagne score, median (IQR)^a^^,^^b^	0 (0 to 1.0)	0 (−1.0 to 1.0)
Gagne category (high, ≥5; low, <5)^a^^,^^c^		
High	376 (2.8)	1987 (0.8)
Low	13 180 (97.2)	262 333 (99.2)
CAN 90-d mortality score, median (IQR)^a^^,^^b^	90 (80 to 98)	50 (30 to 70)
CAN category (high ≥90; low, <90)^a^^,^^c^		
High	9152 (67.5)	41 060 (15.5)
Low	4404 (32.5)	223 260 (84.5)
Cardiometabolic conditions^c^		
History of chronic kidney disease	4278 (31.6)	39 728 (15.0)
History of diabetes	6338 (46.8)	110 593 (41.8)
History of heart failure	3983 (29.4)	27 954 (10.6)
History of hypertension	11 847 (87.4)	233 017 (88.2)
History of ischemic heart disease	4788 (35.3)	55 893 (21.2)
History of peripheral vascular disease	488 (3.6)	3689 (1.4)
History of stroke	2615 (19.3)	23 136 (8.8)
No. of cardiometabolic conditions per patient, median (IQR)	2 (1 to 3)	2 (1 to 2)

Abbreviations: ADI, Area Deprivation Index; CAN, Care Assessment Needs.

^a^ Age, ADI, and the Gagne and CAN scores were calculated at death for mortality group and at cohort entry for no mortality group.

^b^ Ranges: national ADI, 1 to 100; Gagne score, −2 to 26; CAN score, 1 to 100. The CAN score is a commonly used tool that estimates patient percentile risk of mortality or hospitalization. The Gagne score is a comorbidity score.

^c^ Odds ratios for association of extreme heat events with mortality by Gagne category, CAN category, and cardiometabolic conditions are provided in eTable 6 in Supplement 1.

**Figure 1.**
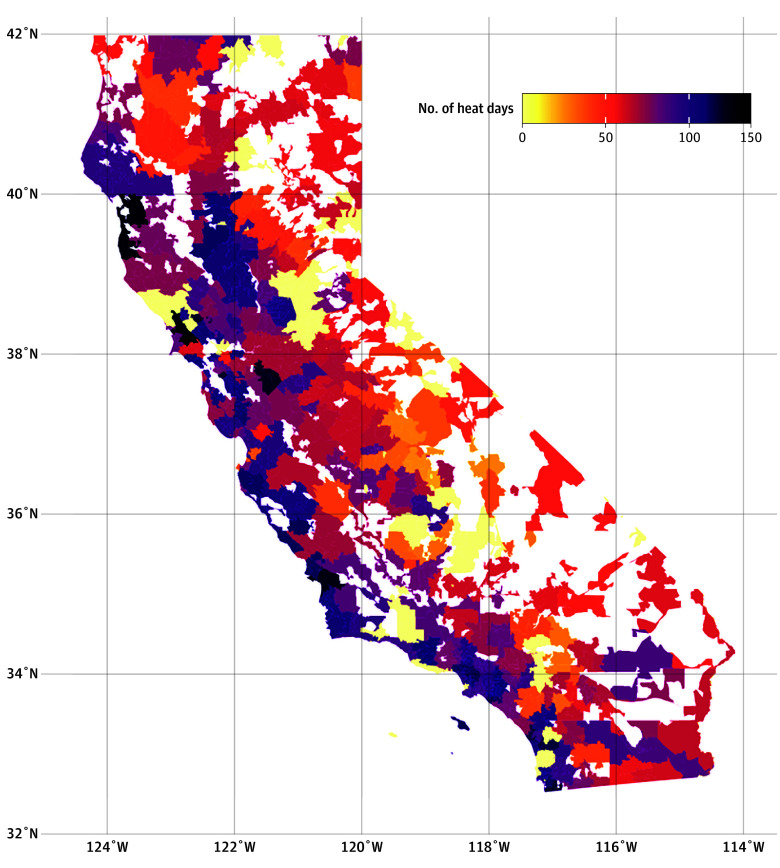
Map of California With Number of Extreme Heat Days Using National Centers for Environmental Information Data and 95th Percentile Threshold White spaces denote regions without unique zip code.

Complete temperature data were available for 3 908 855 of 4 509 464 zip code days (86.7%) and 11 383 veterans during the study period. A total of 492 515 zip code days (12.6%) were extreme heat days using the 90th percentile threshold (eFigure 3 in Supplement 1), 269 711 (6.9%) were extreme heat days using the 95th percentile threshold (Figure 1), and 148 536 (3.8%) were extreme heat days using the 97.5th percentile threshold (eFigure 4 in Supplement 1).

With a 90th percentile threshold, 2-day (OR, 1.10 [95% CI, 1.05-1.16]; *P* < .001), 3-day (OR, 1.10 [95% CI, 1.05-1.15]; *P* < .001), and 4-day (OR, 1.07 [95% CI, 1.02-1.12]; *P* = .005) EHEs were found to be significantly associated with mortality, but not same-day EHEs (OR, 1.05 [95% CI, 0.99-1.12]; *P* = .09) (Table 2). With a 95th percentile threshold, same-day (OR, 1.11 [95% CI, 1.03-1.20]; *P* = .005), 2-day (OR, 1.10 [95% CI, 1.04-1.17]; *P* = .002), 3-day (OR, 1.14 [95% CI, 1.08-1.20]; *P* < .001), and 4-day (OR, 1.11 [95% CI, 1.05-1.17]; *P* < .001) EHEs were found to be significantly associated with mortality. With a 97.5th percentile threshold, same-day (OR, 1.18 [95% CI, 1.07-1.30]; *P* = .001), 2-day (OR, 1.18 [95% CI, 1.10-1.28]; *P* < .001), 3-day (OR, 1.19 [95% CI, 1.11-1.27]; *P* < .001), and 4-day (OR, 1.17 [95% CI, 1.10-1.24]; *P* < .001) EHEs were found to be significantly associated with mortality.

**Table 2.** Unadjusted ORs for Association of EHEs and Mortality Based on Conditional Logistic Regression Models^a^

EHE	90th Percentile		95th Percentile		97.5th Percentile	
	OR (95% CI)	*P* value	OR (95% CI)	*P* value	OR (95% CI)	*P* value
Same day	1.05 (0.99-1.12)	.09	1.11 (1.03-1.20)	.005	1.18 (1.07-1.30)	.001
For 2 d	1.10 (1.05-1.16)	<.001	1.10 (1.04-1.17)	.002	1.18 (1.10-1.28)	<.001
For 3 d	1.10 (1.05-1.15)	<.001	1.14 (1.08-1.20)	<.001	1.19 (1.11-1.27)	<.001
For 4 d	1.07 (1.02-1.12)	.005	1.11 (1.05-1.17)	<.001	1.17 (1.10-1.24)	<.001

Abbreviations: EHE, extreme heat event; OR, odds ratio.

^a^ Sample included 11 383 veterans. Temperature thresholds defined using National Center for Environmental Information data. Table includes associations for same-day EHEs occurring on the same day of mortality through 4-day EHEs lasting from 3 days prior to a patient’s death to the same day as the mortality event. Odds ratios are unadjusted.

**Figure 2.**
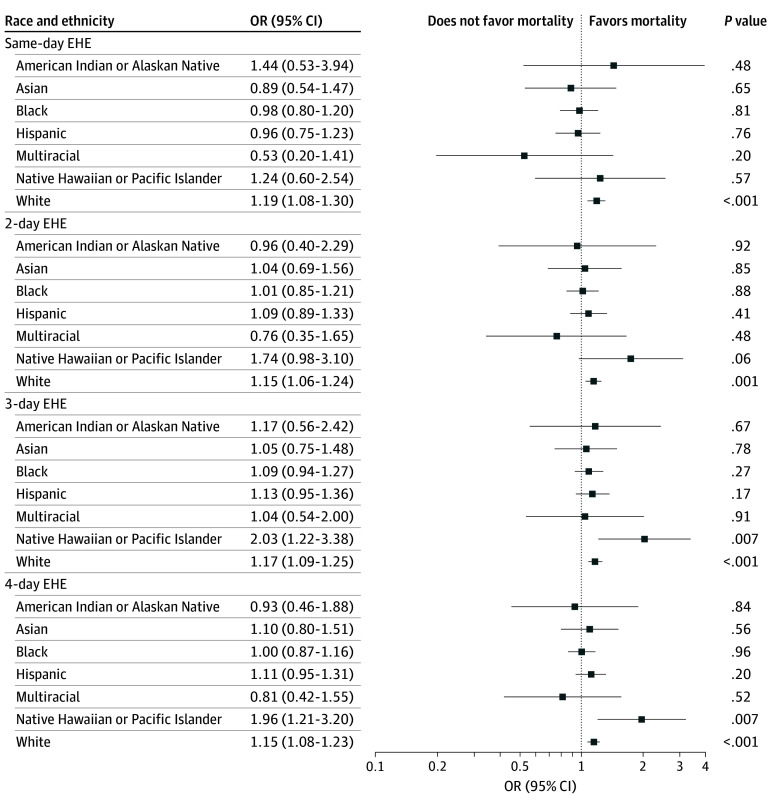
Odds Ratios (ORs) for Associations of Extreme Heat Events (EHEs) at 95th Percentile and Mortality for Race and Ethnicity Subgroups Based on Conditional Logistic Regression Models Temperature thresholds defined using National Center for Environmental Information data. Includes associations for same-day EHEs occurring on the same day of mortality through 4-day EHEs lasting from 3 days prior to a patient’s death to the same day as the mortality event. The *P* value for interaction for all assessments of effect modification was greater than .05 and not shown. American Indian or Alaska Native, n = 75; Asian, n = 282; Black, n = 1467; Hispanic, n = 1122; Native Hawaiian or Other Pacific Islander, n = 122; White, n = 6917; and multiracial, n = 76. Odds ratios are unadjusted.

There was no evidence of significant effect modification among all subgroups of interest at all temperature thresholds, except for Native Hawaiian or Other Pacific Islander veterans compared with White veterans for 4-day heat waves at the 97.5th percentile threshold (eFigure 6 in Supplement 1). At all temperature thresholds, effect estimates for Native Hawaiian or Other Pacific Islander veterans were generally higher compared with White veterans; otherwise, there were no substantial variations in effect estimates by race and ethnicity (Figure 2; eFigures 5 and 6 in Supplement 1).

**Figure 3.**
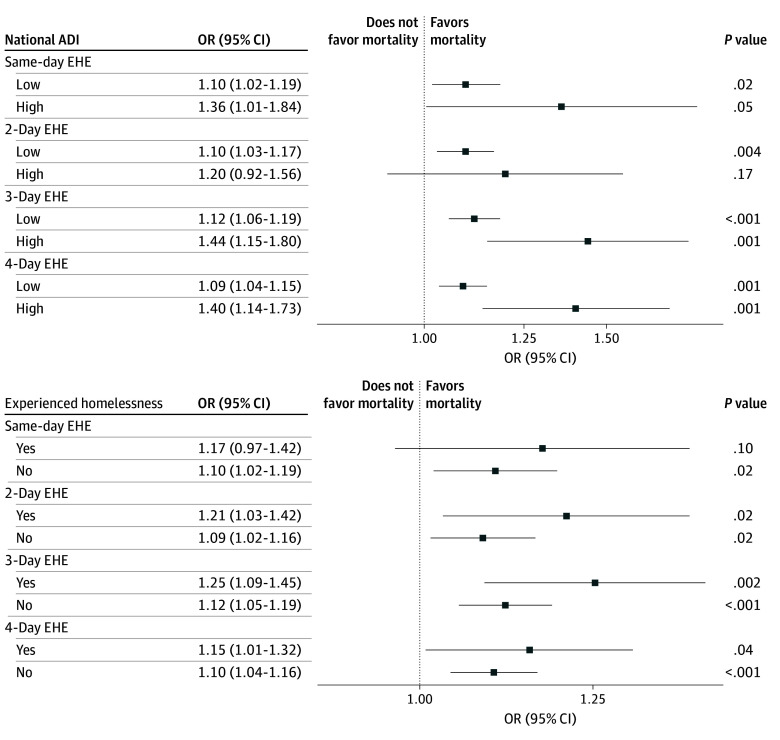
Odds Ratios (ORs) for Associations of Extreme Heat Events (EHEs) at 95th Percentile and Mortality for National Area Deprivation Index (ADI) and Homeless-Experienced Subgroups Based on Conditional Logistic Regression Models Temperature thresholds defined using National Center for Environmental Information data. Includes associations for same-day EHEs occurring on the same day of mortality through 4-day EHEs lasting from 3 days prior to a patient’s death to the same day as the mortality event. A high national ADI is 75 or greater. The *P* value for interaction for all assessments of effect modification was greater than .05 and not shown. High ADI, n = 688; low ADI, n = 10 695; experienced homelessness, n = 1700; did not experience homelessness, n = 9683. Odds ratios are unadjusted.

Generally, effect estimates for the highest quartile ADI were greater than for the lower quartile ADIs. For example, at the 95th percentile threshold, for high vs lower ADI neighborhoods, ORs for 3-day EHEs were 1.44 (95% CI, 1.15-1.80) vs 1.12 (95% CI, 1.06-1.19). At 95th and 97.5th percentile thresholds, ORs for all EHE durations were at least 1.20 (95% CI, 0.92-1.56) and as high as 1.58 (95% CI, 1.22-2.04) for 4-day EHEs at the 97.5th percentile threshold (Figure 3; eFigure 6 in Supplement 1). For homelessness status, at all thresholds, effect estimates for those experiencing homelessness were generally greater compared with those not experiencing homelessness. For example, at the 95th percentile threshold, for veterans experiencing homelessness compared with those who did not, ORs for 3-day EHEs were 1.25 (95% CI, 1.09-1.45) vs 1.12 (95% CI, 1.05-1.19). At the 97.5th percentile threshold, the ORs for veterans experiencing homelessness were at least 1.29 (95% CI, 1.05-1.58; *P* = .01) and as high as 1.36 (95% CI, 1.07-1.73; *P* = .01) for all EHE durations (eFigure 5 in Supplement 1).

### Sensitivity Analyses

Using conditional Poisson or quasi-Poisson regression models resulted in similar findings to conditional logistic regression models, although rate ratios at the 97.5th percentile threshold were not as high (eTable 2 in Supplement 1). Similarly, there was no substantial variation by race and ethnicity, and effect estimates for high ADI and those experiencing homelessness were generally greater than lower ADI and those not experiencing homelessness, respectively (eTable 3 in Supplement 1).

When using zip codes for which we had nonmissing NCEI normal temperatures, findings were similar to the primary analysis; however, effect estimates were generally higher (eTables 4 and 5 in Supplement 1). Odds ratios for highest quartile ADI were as high as 1.67 (95% CI, 1.06-2.62) for same-day EHEs at the 95th percentile and 1.74 (95% CI, 1.10-2.77) for 3-day EHEs at the 97.5th percentile. At the 95th percentile, the OR for experiencing homelessness for same-day EHEs was 1.55 (95% CI, 1.11-1.88), and at the 97.5th percentile, the OR for experiencing homelessness for same-day EHEs was 1.56 (95% CI, 1.10-2.20).

## Discussion

We found that EHEs were associated with mortality among California veterans with common cardiometabolic disease, with an estimated increased odds of mortality of 10% to 18%, depending on the temperature threshold used to define EHEs and the EHE duration. Although our study sample size was inadequate to detect significant effect modification by subgroups, veterans in high ADI neighborhoods had higher odds of death compared with those residing in lower ADI neighborhoods. For veterans who have experienced homelessness, effect estimates were generally greater, reaching as high as 36% increased odds of mortality using a 97.5th percentile threshold. Our primary findings were robust to different modeling approaches. Overall, our analysis suggests that EHEs are associated with risk of mortality among veterans with common cardiometabolic comorbidities and possibly associated with a higher risk among those from socioeconomically vulnerable groups.

It is physiologically plausible that EHEs are associated with excess mortality among veterans with cardiometabolic disease. Excess heat is thought to lead to morbidity and mortality through adaptive physiologic mechanisms, which ultimately lead to a mismatch in myocardial oxygen consumption and supply in vulnerable individuals, raising the risk for cardiac ischemia, kidney injury, and a hypercoagulable state.^14,15,47,65,67^ Effect estimates seemed to decrease slightly for 4-day heat waves compared with 2- and 3-day heat waves, possibly due to veterans seeking assistance during prolonged heat waves. Our findings are in keeping with other studies that have demonstrated increased risk of mortality during EHEs, although no prior studies have specifically investigated all-cause mortality among veterans with cardiometabolic disease,^68,69^ and are likely generalizable to nonveterans with underlying cardiometabolic disease.

In our exploratory analyses, we detected substantially greater effect estimates for Native Hawaiian or Other Pacific Islander veterans compared with White veterans despite a small sample size of Native Hawaiian or Other Pacific Islander veterans and a similar distribution of comorbidities in this group compared with White veterans. Otherwise, we did not detect substantial and consistent variation in effect estimates by race and ethnicity, possibly because of the VA’s care delivery paradigm, which offers low barriers to access for eligible beneficiaries^70^ and makes purposeful efforts to address health-related social factors via screening and interventions.^71^ Although there was no significant effect modification, we detected that for veterans experiencing homelessness, effect estimates were generally higher compared with nonhomeless veterans, suggesting that despite VA access, this group remains profoundly vulnerable to EHEs. This could be due to lack of adequate shelter and overexposure to heat during EHEs or other risk factors with relatively high prevalence in this population, including substance use disorders and psychiatric disorders. The higher mortality trend among veterans in high ADI, compared with lower ADI, neighborhoods could be due to the built environment (eg, urban heat effects, limited access to health services) or individual economic vulnerability leading to limited air conditioning access during extreme heat. Further dedicated research that compares outcomes during EHEs between veterans with and veterans without indicators of socioeconomic vulnerability is warranted.

Prominent national and international public health agencies have encouraged health care systems, in coordination with governmental agencies and community partners, to develop heat response plans to prevent heat-related morbidity, mortality, and acute care use.^72^ Such plans could include heat warning systems and preventive services that reduce downstream EHE-associated morbidity, including designation of cooling centers, financial assistance for the provision of air conditioning, and peer support.^72^ Heat alert systems have been associated with reduced morbidity and mortality during EHEs.^73,74,75,76,77^ By using a variety of temperature thresholds, EHE duration metrics, and patient-level sociodemographic data, our study demonstrates how the association between EHE and mortality varies depending on the desired metric and subgroup. These findings can inform the development of heat-response plans within and outside the VA that incorporate anticipatory alerts directed at socioeconomically vulnerable subgroups based on forecasted EHE severity and length. For example, the VA’s Office of Emergency Management developed a Vulnerable Patient Care, Access and Response in Emergency program to provide guidance and supportive technologies for vulnerable patients during weather-related emergencies,^78^ which could be adapted for use during heat advisories.

### Limitations

This study has some limitations. First, we assumed that the patient address in the CDW was accurate and that patients were at (or near) their home zip code on a given day, although this may not have been the case. Second, we had no measure of indoor heat exposure, as patients may have had access to air conditioning that would have reduced exposure to outdoor heat. Third, our definition of homelessness may not have encompassed all veterans experiencing homelessness.^79^ Also, we included veterans enrolled in homeless programs, including transitional housing programs. From our data, we cannot determine shelter status. It is possible that for veterans experiencing unsheltered homelessness, the association of EHE with mortality is greater than our estimates. Fourth, our study overlaps with the acute phase of the COVID-19 pandemic. However, we would expect veterans to be more inclined to be indoors and not as exposed to outdoor heat, which may bias our findings toward the null. Furthermore, by using a time-stratified design, we could account for secular trends in mortality among our cohort. Fifth, while our time-stratified study design accounts for time-invariant confounders and long-term trends, it cannot account for shorter-term time-variant factors, such as acute changes to clinical status; we would expect these to be nondifferential and potentially bias our findings toward the null.^62^ Sixth, our findings may not be generalizable to veterans outside California and are likely not fully generalizable to non-VA users given differences in VA care delivery compared with health care available to the general US population.

## Conclusions

In this case-crossover study of California veterans with cardiometabolic disease, we found that EHEs were associated with greater mortality for veterans with common cardiometabolic diseases and that this association may be amplified among veterans residing in economically underserved neighborhoods and veterans experiencing homelessness. Given the projected increase in EHEs as global mean temperatures increase, the VA and other integrated health care systems must develop heat preparedness and response plans to protect people from heat-related morbidity and mortality.

## Notes

Supplement 1. **eTable 1.** Cardiometabolic Conditions Based on *International Classification of Disease, Tenth Revision* (*ICD-10*) Codes **eFigure 1.** Sample Flow Chart of California Veterans With Cardiometabolic Disease Between Fiscal Years 2016-2021 **eFigure 2.** Population Density by California County Among California Veterans With Cardiometabolic Disease Between Fiscal Years 2016-2021 Who Experienced Mortality **eFigure 3.** Map of California With Number of Extreme Heat Days Using National Centers for Environmental Information Data and 90^th^ Percentile Threshold **eFigure 4.** Map of California With Number of Extreme Heat Days Using National Centers for Environmental Information Data and 97.5^th^ Percentile Threshold **eFigure 5.** Odds Ratios for Association of Extreme Heat Events at 90^th^ Percentile Threshold and Mortality for A) Race, Ethnicity, B) National Area Deprivation Index (ADI) and C) Homeless Status Based on Conditional Logistic Regression Models **eFigure 6.** Odds Ratios for Association of Extreme Heat Events at 97.5^th^ Percentile Threshold and Mortality for A) Race, Ethnicity, B) National Area Deprivation Index (ADI) and C) Homeless Status Based on Conditional Logistic Regression Models **eTable 2.** Odds Ratios for Association of Extreme Heat Events and Mortality Based on Conditional Poisson Regression Models at Temperature Thresholds Defined Using National Center for Environmental Information Data **eTable 3.** Rate Ratios (RRs) for Association of Extreme Heat Events and Mortality for Race, Ethnicity, Area Deprivation Index and Homeless Status Based on Conditional Poisson/Quasi-Poisson Regression Models at Temperature Thresholds Defined Using National Center for Environmental Information Data Aggregated by California Climate Zone **eTable 4.** Odds Ratios for Association of Extreme Heat Events and Mortality Based on Conditional Logistic Regression Models at Temperature Thresholds Defined Using National Center for Environmental Information Data From Zip Codes With Complete Temperature Data **eTable 5.** Odds Ratios for Association of Extreme Heat Events and Mortality for Race, Ethnicity, Area Deprivation Index and Homeless Status Based on Conditional Logistic Regression Models at Temperature Thresholds Defined Using National Center for Environmental Information Data From Zip Codes With Complete Temperature Data **eTable 6.** Odds Ratio for Association of Extreme Heat Events and Mortality for Gagne Score, Care Assessment Needs (CAN) Score and Cardiometabolic Condition Based on Conditional Logistic Regression Models at Temperature Thresholds Defined Using National Center for Environmental Information Data

Supplement 2.
Data Sharing Statement
