# Supplementary material for: Extreme Heat, Social Factors, and Mortality Among California Veterans With Cardiometabolic Disease
Source: JAMA Netw Open. 2025 Nov 25;8(11):e2545524. doi: 10.1001/jamanetworkopen.2025.45524 (PMC12648351; doi:10.1001/jamanetworkopen.2025.45524)
Supplement: Supplement 1. — eTable 1. Cardiometabolic Conditions Based on International Classification of Disease, Tenth Revision (ICD-10) Codes eFigure 1. Sample Flow Chart of California Veterans With Cardiometabolic Disease Between Fiscal Years 2016-2021 eFigure 2. Population Density by California County Among California Veterans With Cardiometabolic Disease Between Fiscal Years 2016-2021 Who Experienced Mortality eFigure 3. Map of California With Number of Extreme Heat Days Using National Centers for Environmental Information Data and 90th Percentile Threshold eFigure 4. Map of California With Number of Extreme Heat Days Using National Centers for Environmental Information Data and 97.5th Percentile Threshold eFigure 5. Odds Ratios for Association of Extreme Heat Events at 90th Percentile Threshold and Mortality for A) Race, Ethnicity, B) National Area Deprivation Index (ADI) and C) Homeless Status Based on Conditional Logistic Regression Models eFigure 6. Odds Ratios for Association of Extreme Heat Events at 97.5th Percentile Threshold and Mortality for A) Race, Ethnicity, B) National Area Deprivation Index (ADI) and C) Homeless Status Based on Conditional Logistic Regression Models eTable 2. Odds Ratios for Association of Extreme Heat Events and Mortality Based on Conditional Poisson Regression Models at Temperature Thresholds Defined Using National Center for Environmental Information Data eTable 3. Rate Ratios (RRs) for Association of Extreme Heat Events and Mortality for Race, Ethnicity, Area Deprivation Index and Homeless Status Based on Conditional Poisson/Quasi-Poisson Regression Models at Temperature Thresholds Defined Using National Center for Environmental Information Data Aggregated by California Climate Zone eTable 4. Odds Ratios for Association of Extreme Heat Events and Mortality Based on Conditional Logistic Regression Models at Temperature Thresholds Defined Using National Center for Environmental Information Data From Zip Codes With Complete Temperature Data eTable 5. Odds [file jamanetwopen-e2545524-s001.pdf]

Supplementary Online Content

Shannon EM, Chen L, Yuan A, et al. Extreme heat, social factors, and mortality among California veterans with cardiometabolic disease. *JAMA Netw Open.* 2025;8(11):e2545524. doi:10.1001/jamanetworkopen.2025.45524

eTable 1. Cardiometabolic Conditions Based on *International Classification of Disease, Tenth Revision* (*ICD-10*) Codes
eFigure 1. Sample Flow Chart of California Veterans With Cardiometabolic Disease Between Fiscal Years 2016-2021
eFigure 2. Population Density by California County Among California Veterans With Cardiometabolic Disease Between Fiscal Years 2016-2021 Who Experienced Mortality
Figure 3. Map of California With Number of Extreme Heat Days Using National Centers for Environmental Information Data and 90th Percentile Threshold
eFigure 4. Map of California With Number of Extreme Heat Days Using National Centers for Environmental Information Data and 97.5th Percentile Threshold
eFigure 5. Odds Ratios for Association of Extreme Heat Events at 90th Percentile Threshold and Mortality for A) Race, Ethnicity, B) National Area Deprivation Index (ADI) and C) Homeless Status Based on Conditional Logistic Regression Models
eFigure 6. Odds Ratios for Association of Extreme Heat Events at 97.5th Percentile Threshold and Mortality for A) Race, Ethnicity, B) National Area Deprivation Index (ADI) and C) Homeless Status Based on Conditional Logistic Regression Models
eTable 2. Odds Ratios for Association of Extreme Heat Events and Mortality Based on Conditional Poisson Regression Models at Temperature Thresholds Defined Using National Center for Environmental Information Data
eTable 3. Rate Ratios (RRs) for Association of Extreme Heat Events and Mortality for Race, Ethnicity, Area Deprivation Index and Homeless Status Based on Conditional Poisson/Quasi-Poisson Regression Models at Temperature Thresholds Defined Using National Center for Environmental Information Data Aggregated by California Climate Zone
eTable 4. Odds Ratios for Association of Extreme Heat Events and Mortality Based on Conditional Logistic Regression Models at Temperature Thresholds Defined Using National Center for Environmental Information Data From Zip Codes With Complete Temperature Data
eTable 5. Odds Ratios for Association of Extreme Heat Events and Mortality for Race, Ethnicity, Area Deprivation Index and Homeless Status Based on Conditional Logistic Regression Models at Temperature Thresholds Defined Using National Center for Environmental Information Data From Zip Codes With Complete Temperature Data
eTable 6. Odds Ratio for Association of Extreme Heat Events and Mortality for Gagne Score, Care Assessment Needs (CAN) Score and Cardiometabolic Condition Based on Conditional Logistic Regression Models at Temperature Thresholds Defined Using National Center for Environmental Information Data

This supplementary material has been provided by the authors to give readers additional information about their work.

© 2025 Shannon EM et al. *JAMA Network Open.*

eTable 1. Cardiometabolic Conditions Based on *International Classification of Disease, Tenth Revision* (*ICD-10*) Codes

Condition	ICD-10 Code	Condition Name
Hypertension	I10	Essential (primary) hypertension
	I11	Hypertensive heart disease
	I12	Hypertensive chronic kidney disease
	I13	Hypertensive heart and chronic kidney disease
	I15	Secondary hypertension
	I16	Hypertensive crisis
Diabetes Mellitus	E10-E14	Typ1, Type 2, other unspecified
Stroke	I60-69	Cerebrovascular disease
Heart Failure	I42	Cardiomyopathy
	I43	Cardiomyopathy in diseases classified elsewhere
	I50	Heart failure
Ischemic heart disease	I25	Chronic ischemic heart disease
Peripheral vascular disease	I70.2	Atherosclerosis of native arteries of the extremities
	I70.3	Atherosclerosis of unspecified type of bypass graft(s) of the extremities
	I70.4	Atherosclerosis of autologous vein bypass graft(s) of the extremities
	I70.5	Atherosclerosis of non-autologous biological bypass graft(s) of the extremities
	I70.6	Atherosclerosis of nonbiological bypass graft(s) of the extremities
	I70.7	Atherosclerosis of other type of bypass graft(s) of the extremities
Chronic Kidney Disease	N18	Chronic kidney disease

© 2025 Shannon EM et al. *JAMA Network Open.*

eFigure 1. Sample Flow Chart of California Veterans With Cardiometabolic Disease Between Fiscal Years 2016-2021. Note: cardiometabolic conditons include: hypertension, diabetes mellitus, ischemic heart disease, congestive heart failure, chronic kidney disease, stroke and peripheral arterial disease.

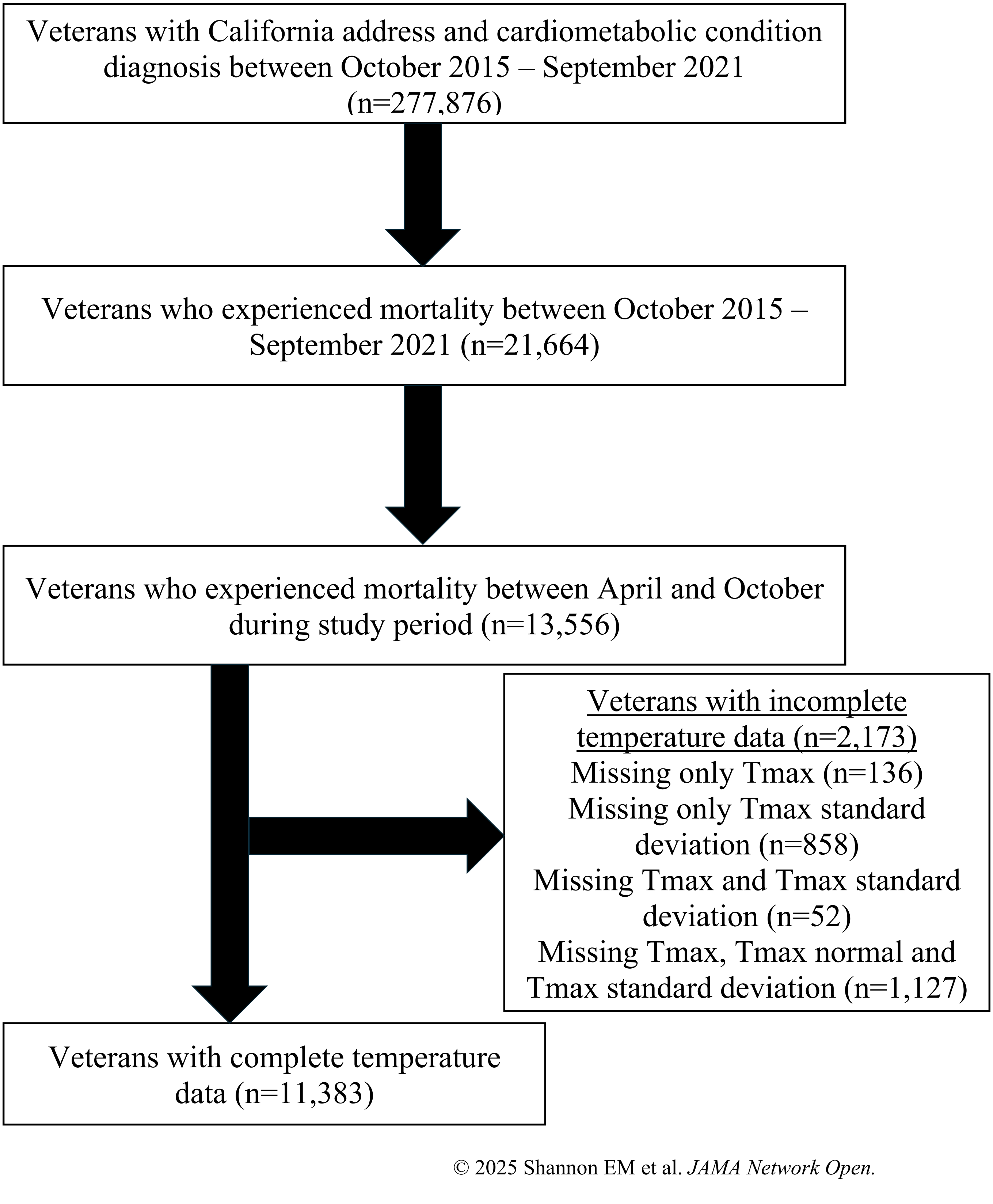

© 2025 Shannon EM et al. *JAMA Network Open.*

eFigure 2. Population Density by California County Among California Veterans With Cardiometabolic Disease Between Fiscal Years 2016-2021 Who Experienced Mortality

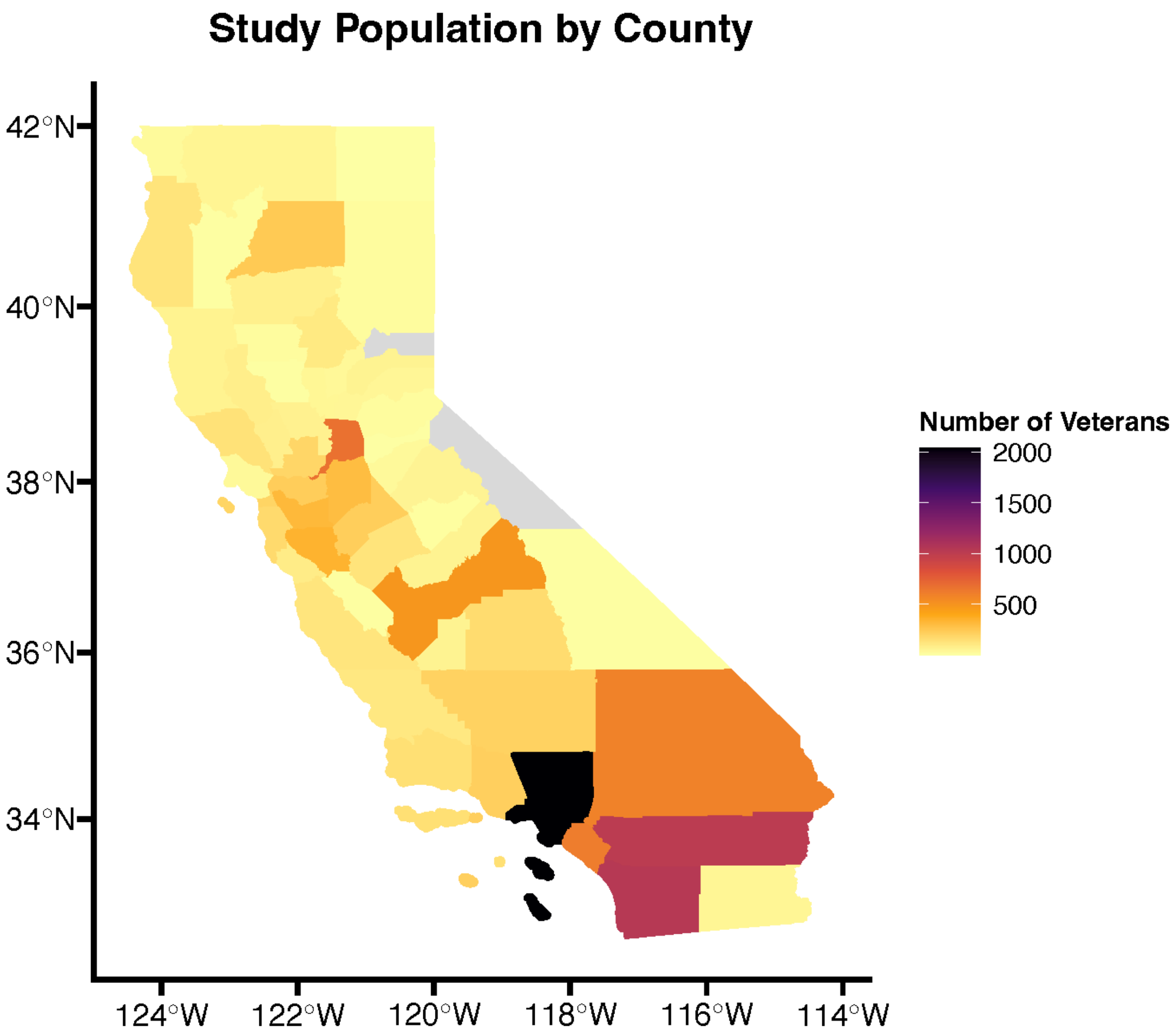

© 2025 Shannon EM et al. *JAMA Network Open.*

Figure 3. Map of California With Number of Extreme Heat Days Using National Centers for Environmental Information Data and 90th Percentile Threshold. Note: white spaces denote regions without unique zip code.

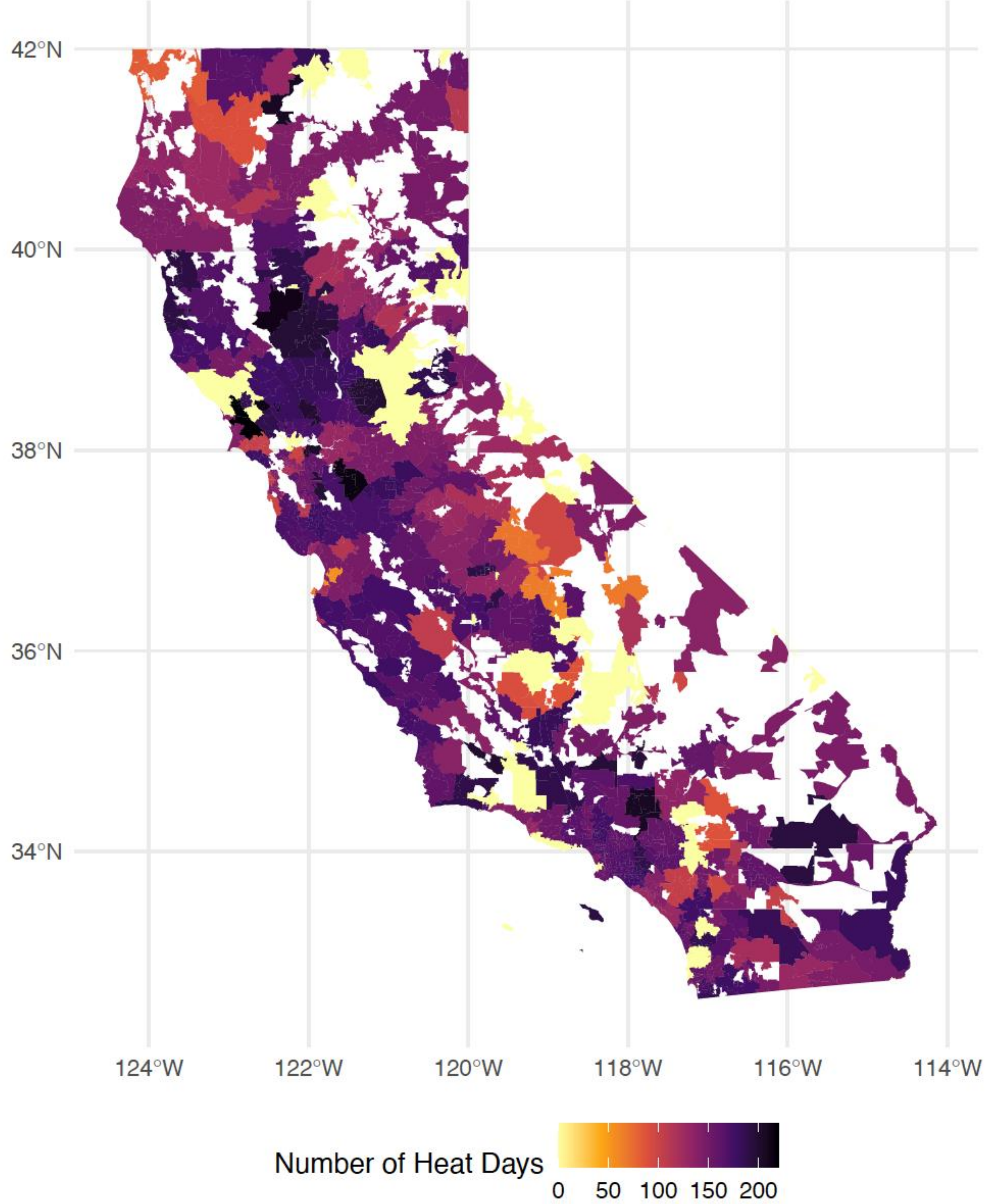

© 2025 Shannon EM et al. *JAMA Network Open.*

eFigure 4. Map of California With Number of Extreme Heat Days Using National Centers for Environmental Information Data and 97.5th Percentile Threshold. Note: white spaces denote regions without unique zip code.

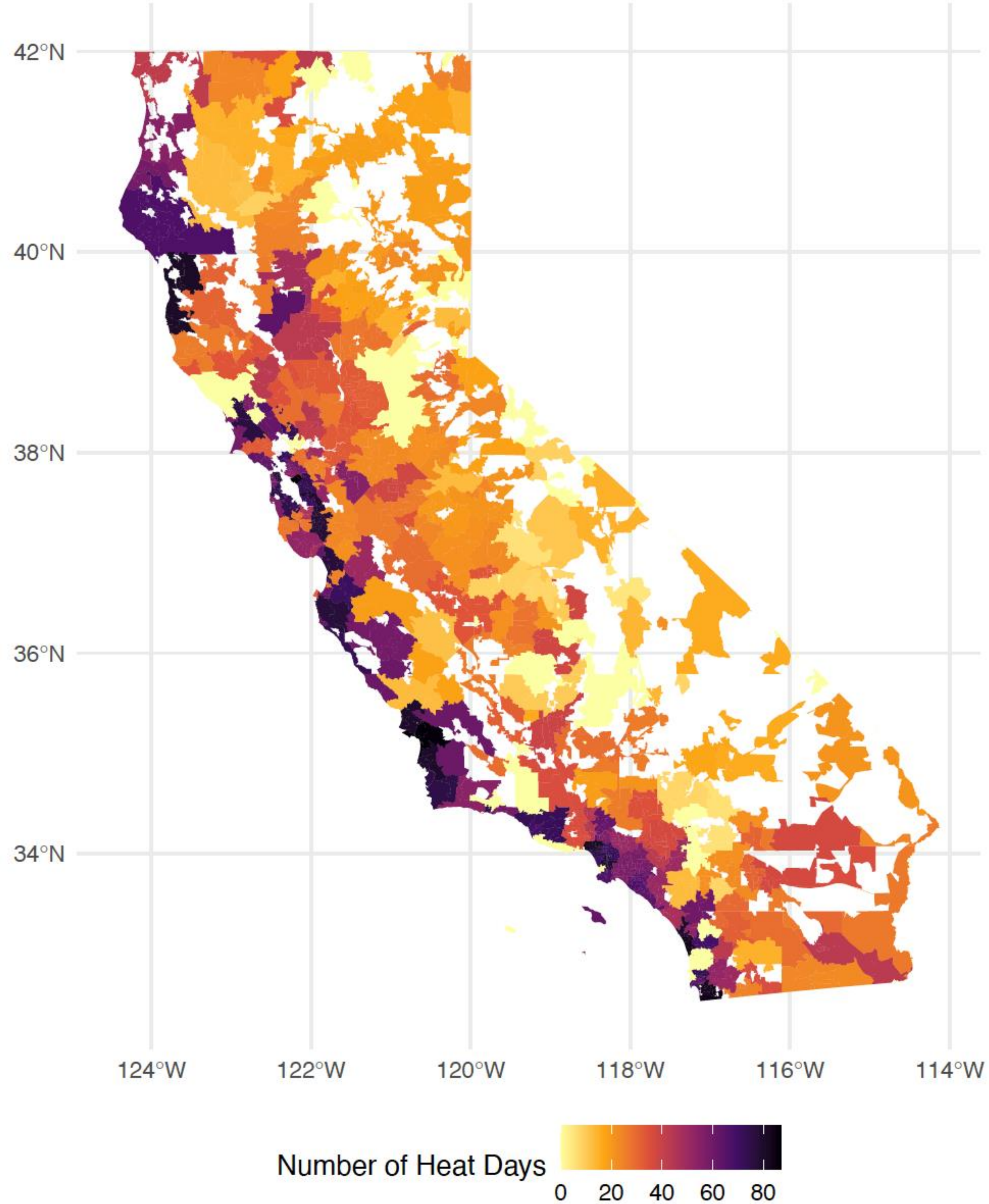

© 2025 Shannon EM et al. *JAMA Network Open.*

eFigure 5. Odds Ratios for Association of Extreme Heat Events at 90th Percentile Threshold and Mortality for A) Race, Ethnicity, B) National Area Deprivation Index (ADI) and C) Homeless Status Based on Conditional Logistic Regression Models

Race and ethnicity	OR (95% CI)	p-value
AIAN	1.34 (0.65-2.75)	0.43
Asian	0.86 (0.57-1.29)	0.47
Black	0.93 (0.79-1.10)	0.42
Hispanic	1.06 (0.87-1.28)	0.57
Multi-Race	0.56 (0.26-1.21)	0.14
NHPI	1.09 (0.63-1.98)	0.77
White	1.09 (1.01-1.17)	0.03
AIAN	1.31 (0.71-2.42)	0.38
Asian	0.98 (0.70-1.37)	0.91
Black	0.95 (0.82-1.09)	0.45
Hispanic	1.10 (0.94-1.30)	0.25
Multi-Race	0.86 (0.46-1.61)	0.64
NHPI	1.70 (1.07-2.70)	0.03
White	1.15 (1.08-1.22)	<0.001
AIAN	1.31 (0.74-2.32)	0.36
Asian	0.97 (0.72-1.31)	0.84
Black	1.02 (0.90-1.16)	0.75
Hispanic	1.12 (0.96-1.30)	0.14
Multi-Race	0.96 (0.55-1.70)	0.90
NHPI	1.54 (1.01-2.36)	0.05
White	1.13 (1.07-1.20)	<0.001
AIAN	1.02 (0.58-1.80)	0.95
Asian	1.06 (0.80-1.41)	0.68
Black	0.92 (0.82-1.05)	0.21
Hispanic	1.09 (0.95-1.26)	0.24
Multi-Race	0.83 (0.47-1.45)	0.50
NHPI	1.35 (0.89-2.05)	0.16
White	1.11 (1.05-1.18)	<0.001

A

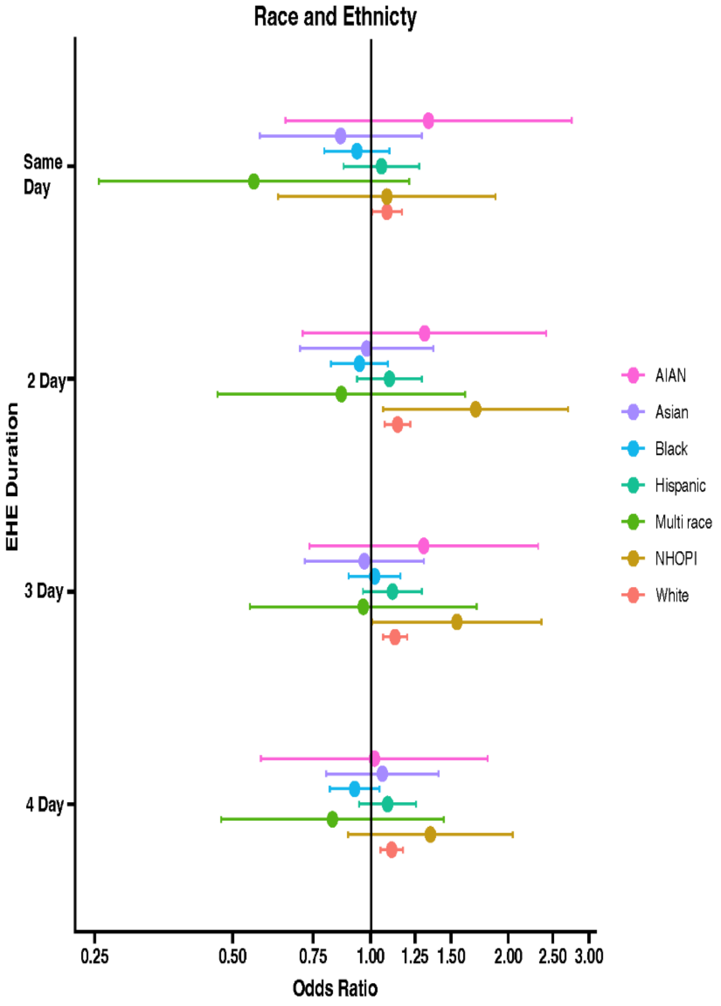

© 2025 Shannon EM et al. *JAMA Network Open.*

National ADI	OR (95% CI)	p-value
Low	1.03 (0.95-1.10)	0.51
High	1.13 (0.89-1.43)	0.31
Low	1.09 (1.04-1.15)	<0.001
High	1.18 (0.96-1.44)	0.11
Low	1.09 (1.03-1.14)	0.00
High	1.31 (1.09-1.58)	0.01
Low	1.06 (1.01-1.10)	0.02
High	1.27 (1.06-1.52)	0.01

B

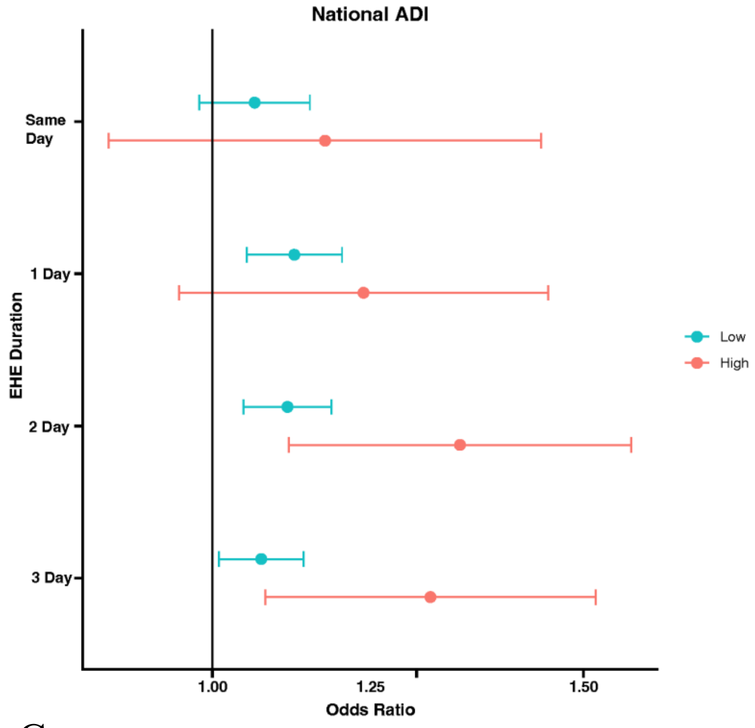

Homeless Experienced	OR (95% CI)	p-value
Yes	1.13 (0.97-1.31)	0.12
No	1.04 (0.98-1.11)	0.23
Yes	1.19 (1.05-1.36)	0.008
No	1.08 (1.03-1.14)	0.004
Yes	1.22 (1.08-1.37)	0.001
No	1.08 (1.03-1.14)	0.004
Yes	1.14 (1.01-1.28)	0.03
No	1.06 (1.01-1.11)	0.03

C

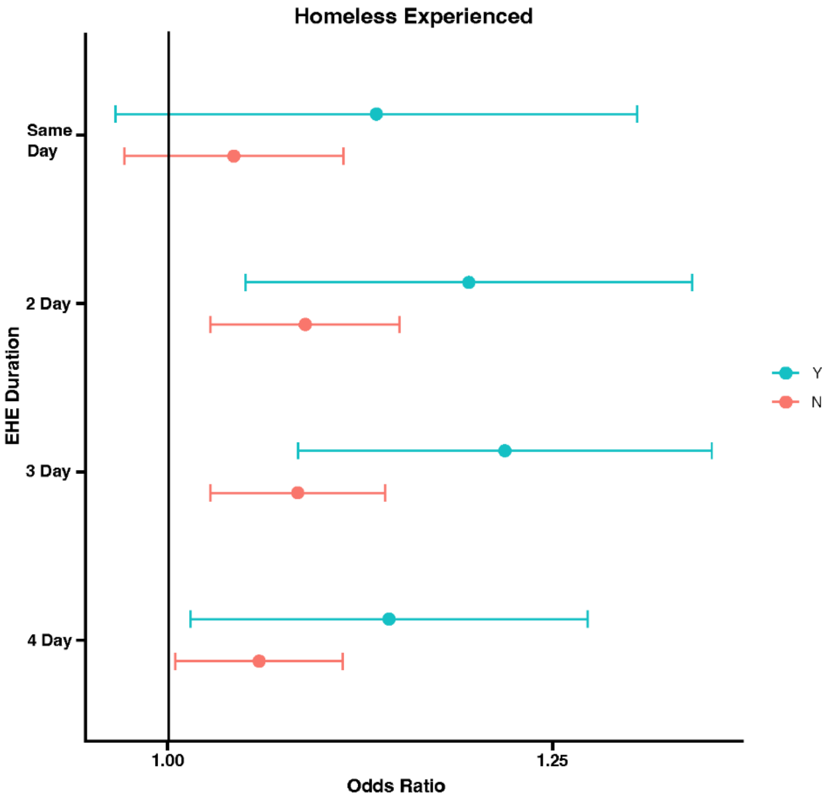

© 2025 Shannon EM et al. *JAMA Network Open.*

Note: Temperature thresholds defined using National Center for Environmental Information data. Includes associations for single-day EHEs occurring on the same day of mortality through 4-day EHEs lasting from 3 days prior to a patient's death to the same day as the mortality event. High National ADI ≥ 75. P-for-interaction for all assessments of effective modification >0.05 and not shown. AIAN n=75, Asian n=282, Black n=1,467, Hispanic n=1,122, Multi-Race n=76, NHPI n=122, White n=6,917. High ADI n=688, Low ADI n=10,695, homeless-experienced n=1,700, non-homeless experienced n=9,683. Abbreviations. ADI, area deprivation index; AIAN, American Indian Alaska Native; NHPI, Native Hawaiian Pacific Islander; CI, confidence interval; OR, odds ratio.

© 2025 Shannon EM et al. *JAMA Network Open.*

eFigure 6. Odds Ratios for Association of Extreme Heat Events at 97.5th Percentile Threshold and Mortality for A) Race, Ethnicity, B) National Area Deprivation Index (ADI) and C) Homeless Status Based on Conditional Logistic Regression Models

Race and ethnicity	OR (95% CI)	p-value
AIAN	1.25 (0.31-5.11)	0.76
Asian	0.75 (0.38-1.48)	0.41
Black	1.04 (0.80-1.34)	0.78
Hispanic	1.26 (0.92-1.72)	0.15
Multi-Race	0.54 (0.16-1.86)	0.33
NHPI	1.44 (0.63-3.28)	0.39
White	1.24 (1.10-1.40)	0.00
AIAN	0.63 (0.18-2.26)	0.48
Asian	0.71 (0.40-1.26)	0.24
Black	1.07 (0.86-1.33)	0.54
Hispanic	1.17 (0.90-1.52)	0.23
Multi-Race	0.61 (0.20-1.88)	0.39
NHPI	1.59 (0.80-3.16)	0.19
White	1.28 (1.16-1.41)	<0.001
AIAN	0.84 (0.30-2.35)	0.74
Asian	0.98 (0.63-1.51)	0.93
Black	1.14 (0.95-1.37)	0.17
Hispanic	1.20 (0.95-1.51)	0.12
Multi-Race	0.88 (0.37-2.12)	0.78
NHPI	2.19 (1.21-3.96)	0.01
White	1.23 (1.12-1.34)	<0.001
AIAN	0.62 (0.23-1.71)	0.36
Asian	1.07 (0.73-1.57)	0.74
Black	1.09 (0.92-1.29)	0.33
Hispanic	1.30 (1.06-1.59)	0.01
Multi-Race	0.79 (0.35-1.80)	0.58
NHPI	2.31 (1.32-4.03)	0.003
White	1.20 (1.10-1.30)	<0.001

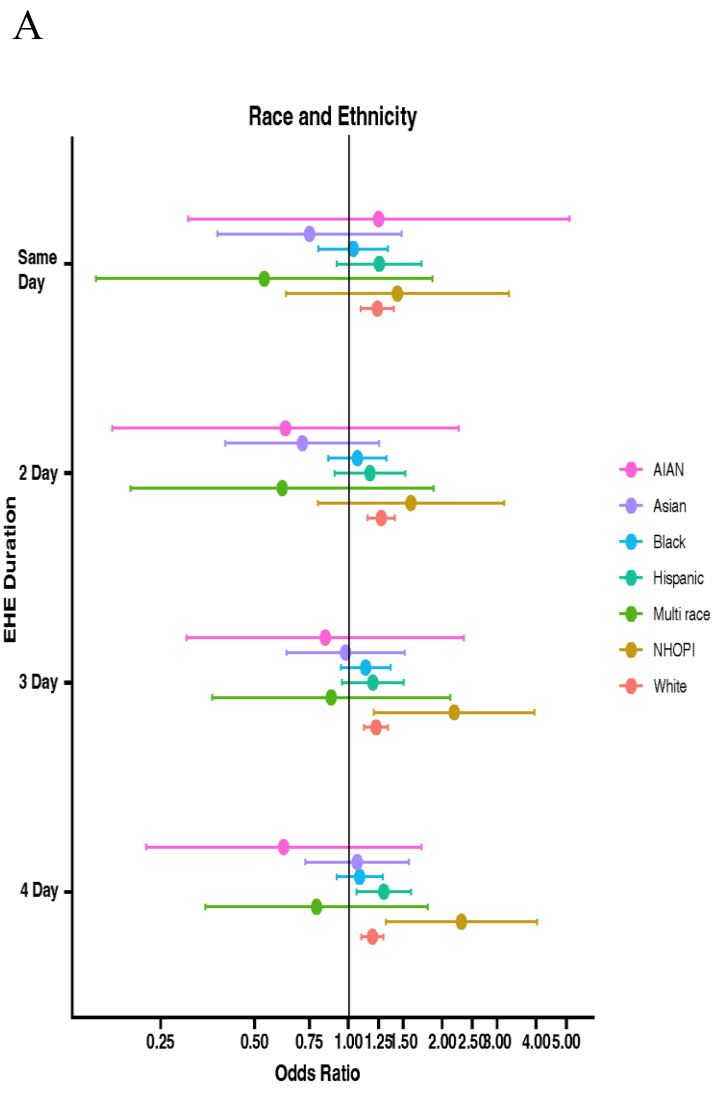

© 2025 Shannon EM et al. *JAMA Network Open.*

National ADI	OR (95% CI)	p-value
Low	1.16 (1.05-1.28)	0.003
High	1.54 (1.04-2.30)	0.03
Low	1.18 (1.09-1.28)	<0.001
High	1.24 (0.89-1.73)	0.214
Low	1.17 (1.09-1.25)	<0.001
High	1.49 (1.12-1.98)	0.006
Low	1.15 (1.08-1.22)	<0.001
High	1.58 (1.22-2.04)	<0.001

B

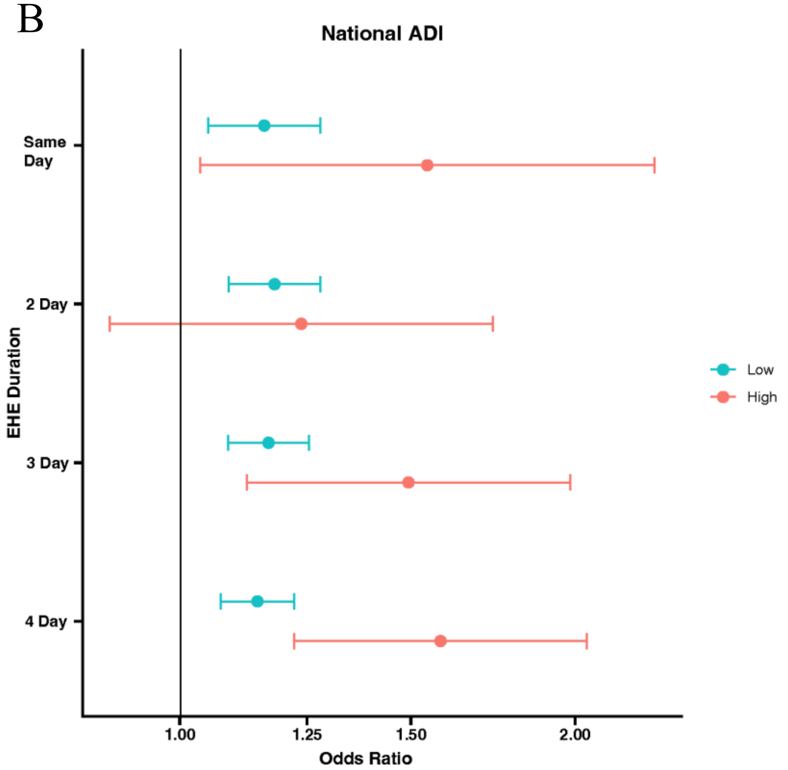

Homeless Experienced	OR (95% CI)	p-value
Yes	1.36 (1.07-1.73)	0.013
No	1.15 (1.04-1.27)	0.009
Yes	1.29 (1.06-1.58)	0.013
No	1.17 (1.07-1.27)	<0.001
Yes	1.35 (1.14-1.61)	0.001
No	1.16 (1.08-1.25)	<0.001
Yes	1.29 (1.10-1.52)	0.002
No	1.15 (1.07-1.23)	<0.001

C

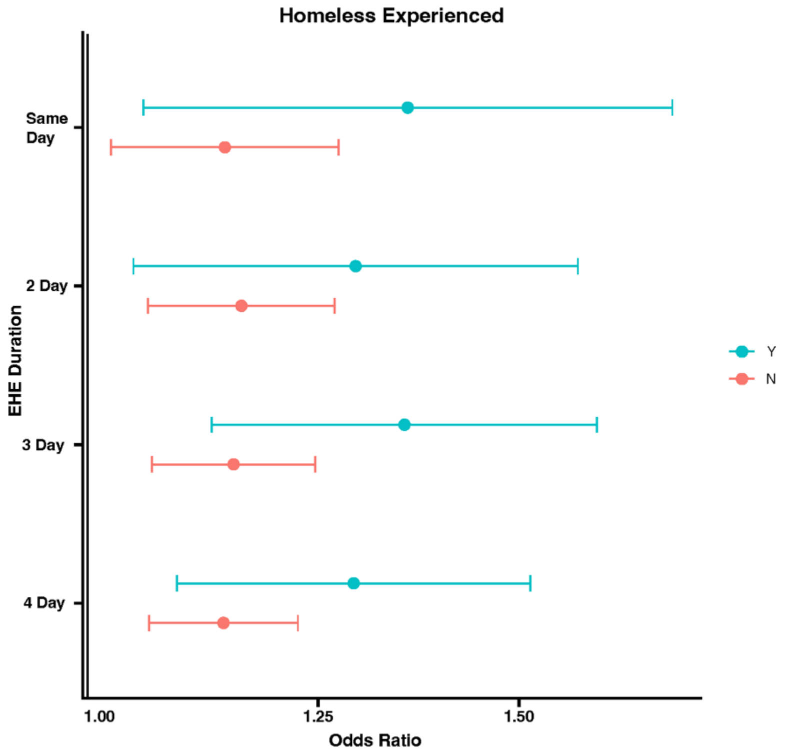

Note: temperature thresholds defined using National Center for Environmental Information data. Includes associations for single-day EHEs occurring on the same day of mortality through 4-day

© 2025 Shannon EM et al. *JAMA Network Open.*

EHEs lasting from 3 days prior to a patient's death to the same day as the mortality event. High National ADI ≥75. P-for-interaction for NHPI v White at 4-day = 0.02, otherwise all assessments of effective modification >0.05 and not shown. . AIAN n=75, Asian n=282, Black n=1,467, Hispanic n=1,122, Multi-Race n=76, NHPI n=122, White n=6,917. High ADI n=688, Low ADI n=10,695, homeless-experienced n=1,700, non-homeless experienced n=9,683. Abbreviations. ADI, area deprivation index; AIAN, American Indian Alaska Native; NHPI, Native Hawaiian Pacific Islander; CI, confidence interval; OR, odds ratio.

© 2025 Shannon EM et al. *JAMA Network Open.*

eTable 2. Odds Ratios for Association of Extreme Heat Events and Mortality Based on Conditional Poisson Regression Models at Temperature Thresholds Defined Using National Center for Environmental Information Data. Includes associations for single-day EHEs occurring on the same day of mortality through 4-day EHEs lasting from 3 days prior to a patient's death to the same day as the mortality event. Note: estimates for conditional quasi-Poisson were identical.

	90th percentile				***95th percentile***				***97.5th percentile***			
	**OR**	**95% CI**		**p-value**	**OR**	**95% CI**		**p-value**	**OR**	**95% CI**		**p-value**
Same-day	1.08	1.02	1.13	0.003	1.08	1.02	1.14	0.006	1.10	1.03	1.18	0.003
2 day	1.08	1.03	1.18	0.001	1.08	1.03	1.14	0.001	1.09	1.03	1.15	0.002
3 day	1.08	1.03	1.13	0.003	1.06	1.01	1.11	0.01	1.07	1.02	1.13	0.008
4 day	1.07	1.02	1.12	0.009	1.11	1.01	1.10	0.03	1.09	1.04	1.14	<0.001

© 2025 Shannon EM et al. *JAMA Network Open.*

eTable 3. Rate Ratios (RRs) for Association of Extreme Heat Events and Mortality for Race, Ethnicity, Area Deprivation Index and Homeless Status Based on Conditional Poisson/Quasi-Poisson Regression Models at Temperature Thresholds Defined Using National Center for Environmental Information Data Aggregated by California Climate Zone. Includes associations for single-day EHEs occurring on the same day of mortality through 4-day EHEs lasting from 3 days prior to a patient's death to the same day as the mortality event. P-for-interaction for all assessments of effective modification >0.05 and not shown. High National ADI ≥75. Abbreviations. ADI, area deprivation index; AIAN, American Indian Alaska Native; CI, confidence interval; NHPI, Native Hawaiian, Pacific Islander; OR, odds ratio

**90th percentile Same-day**	**RR**	**95% CI**		**p-value**
Overall	1.08	1.03	1.13	0.002
High National ADI	1.10	0.88	1.37	0.402
Low National ADI	1.08	1.03	1.14	0.001
Non-Hispanic White	1.06	1.00	1.13	0.046
Non-Hispanic Asian	0.85	0.62	1.19	0.346
Non-Hispanic Black	0.98	0.86	1.12	0.775
Hispanic	1.07	0.92	1.24	0.412
Non-Hispanic NHPI	1.15	0.70	1.87	0.585
Non-Hispanic AIAN	0.97	0.52	1.82	0.933
Non-Hispanic Multiracial	0.78	0.43	1.40	0.404
Has Been Homeless	1.12	1.00	1.27	0.052
Has Never Been Homeless	1.05	1.00	1.11	0.039
**2 day**				
Overall	1.08	1.03	1.13	0.001
High National ADI	1.07	0.87	1.30	0.536
Low National ADI	1.09	1.04	1.14	<0.001
Non-Hispanic White	1.08	1.02	1.15	0.008
Non-Hispanic Asian	0.99	0.74	1.32	0.935
Non-Hispanic Black	1.00	0.89	1.13	0.978
Hispanic	1.06	0.93	1.21	0.411
Non-Hispanic NHPI	1.71	1.12	2.62	0.014
Non-Hispanic AIAN	1.08	0.62	1.86	0.790
Non-Hispanic Multiracial	0.97	0.58	1.59	0.889
Has Been Homeless	1.20	1.08	1.34	0.001
Has Never Been Homeless	1.06	1.01	1.12	0.016
**3 day**				
Overall	1.08	1.03	1.13	0.002
High National ADI	1.19	0.98	1.44	0.084
Low National ADI	1.08	1.03	1.13	0.002
Non-Hispanic White	1.09	1.03	1.16	0.003
Non-Hispanic Asian	1.02	0.79	1.33	0.861

© 2025 Shannon EM et al. *JAMA Network Open.*

Non-Hispanic Black	1.04	0.93	1.16	0.526
Hispanic	1.05	0.92	1.19	0.453
Non-Hispanic NHPI	1.60	1.08	2.35	0.019
Non-Hispanic AIAN	1.18	0.69	1.99	0.547
Non-Hispanic Multiracial	1.01	0.63	1.64	0.955
Has Been Homeless	1.18	1.06	1.30	0.002
Has Never Been Homeless	1.07	1.02	1.12	0.010
**4 day**				
Overall	1.07	1.02	1.12	0.006
High National ADI	1.19	0.99	1.44	0.069
Low National ADI	1.06	1.01	1.11	0.022
Non-Hispanic White	1.10	1.04	1.17	0.002
Non-Hispanic Asian	1.09	0.85	1.40	0.506
Non-Hispanic Black	0.93	0.83	1.04	0.190
Hispanic	1.01	0.89	1.14	0.929
Non-Hispanic NHPI	1.44	0.99	2.11	0.059
Non-Hispanic AIAN	0.94	0.56	1.58	0.813
Non-Hispanic Multiracial	0.87	0.54	1.39	0.562
Has Been Homeless	1.09	0.98	1.21	0.098
Has Never Been Homeless	1.07	1.02	1.13	0.007
**95th percentile**				
**same-day**				
Overall	1.08	1.02	1.14	0.004
High National ADI	1.30	0.96	1.76	0.097
Low National ADI	1.07	1.02	1.13	0.012
Non-Hispanic White	1.11	1.03	1.19	0.004
Non-Hispanic Asian	0.84	0.53	1.34	0.457
Non-Hispanic Black	1.02	0.86	1.20	0.843
Hispanic	1.03	0.84	1.25	0.786
Non-Hispanic NHPI	1.15	0.60	2.23	0.671
Non-Hispanic AIAN	1.30	0.51	3.29	0.587
Non-Hispanic Multiracial	0.71	0.33	1.51	0.373
Has Been Homeless	1.18	1.01	1.38	0.038
Has Never Been Homeless	1.08	1.02	1.14	0.012
**2 day**				
Overall	1.08	1.04	1.14	0.001
High National ADI	1.12	0.86	1.47	0.402
Low National ADI	1.07	1.02	1.13	0.003
Non-Hispanic White	1.10	1.03	1.17	0.002
Non-Hispanic Asian	1.18	0.82	1.70	0.361
Non-Hispanic Black	1.03	0.89	1.19	0.735
Hispanic	1.09	0.92	1.29	0.317

© 2025 Shannon EM et al. *JAMA Network Open.*

Non-Hispanic NHPI	1.66	0.97	2.81	0.064
Non-Hispanic AIAN	0.86	0.38	1.94	0.710
Non-Hispanic Multiracial	0.95	0.50	1.81	0.883
Has Been Homeless	1.12	0.98	1.28	0.092
Has Never Been Homeless	1.08	1.03	1.14	0.002
**3 day**				
Overall	1.06	1.01	1.11	0.009
High National ADI	1.36	1.07	1.72	0.011
Low National ADI	1.05	1.00	1.10	0.042
Non-Hispanic White	1.09	1.03	1.15	0.005
Non-Hispanic Asian	1.21	0.89	1.64	0.233
Non-Hispanic Black	1.07	0.94	1.22	0.315
Hispanic	1.13	0.97	1.31	0.125
Non-Hispanic NHPI	1.91	1.19	3.06	0.007
Non-Hispanic AIAN	1.10	0.55	2.20	0.782
Non-Hispanic Multiracial	1.23	0.71	2.14	0.464
Has Been Homeless	1.18	1.04	1.33	0.010
Has Never Been Homeless	1.06	1.01	1.11	0.020
**4 day**				
Overall	1.05	1.01	1.09	0.023
High National ADI	1.28	1.03	1.60	0.026
Low National ADI	1.04	1.00	1.09	0.072
Non-Hispanic White	1.07	1.02	1.13	0.013
Non-Hispanic Asian	1.24	0.93	1.65	0.148
Non-Hispanic Black	1.01	0.89	1.14	0.872
Hispanic	1.11	0.96	1.28	0.163
Non-Hispanic NHPI	1.85	1.18	2.89	0.007
Non-Hispanic AIAN	0.88	0.45	1.72	0.711
Non-Hispanic Multiracial	0.96	0.55	1.67	0.890
Has Been Homeless	1.10	0.98	1.23	0.113
Has Never Been Homeless	1.05	1.01	1.10	0.028
**97.5th percentile same-day**				
Overall	1.10	1.04	1.17	0.002
High National ADI	1.53	1.01	2.33	0.047
Low National ADI	1.10	1.03	1.18	0.003
Non-Hispanic White	1.13	1.04	1.23	0.004
Non-Hispanic Asian	0.77	0.42	1.41	0.391
Non-Hispanic Black	1.07	0.87	1.32	0.530
Hispanic	1.22	0.95	1.58	0.122
Non-Hispanic NHPI	1.30	0.62	2.76	0.491
Non-Hispanic AIAN	1.25	0.33	4.70	0.741

© 2025 Shannon EM et al. *JAMA Network Open.*

Non-Hispanic Multiracial	0.74	0.28	1.95	0.542
Has Been Homeless	1.35	1.11	1.64	0.003
Has Never Been Homeless	1.10	1.03	1.18	0.006
**2 day**				
Overall	1.09	1.03	1.15	0.002
High National ADI	1.27	0.89	1.80	0.190
Low National ADI	1.09	1.03	1.15	0.003
Non-Hispanic White	1.12	1.05	1.21	0.001
Non-Hispanic Asian	0.77	0.47	1.27	0.305
Non-Hispanic Black	1.14	0.96	1.35	0.142
Hispanic	1.25	1.01	1.54	0.037
Non-Hispanic NHPI	1.45	0.78	2.71	0.240
Non-Hispanic AIAN	0.63	0.19	2.09	0.450
Non-Hispanic Multiracial	0.87	0.35	2.15	0.756
Has Been Homeless	1.21	1.02	1.43	0.025
Has Never Been Homeless	1.09	1.03	1.15	0.005
**3 day**				
Overall	1.07	1.02	1.12	0.006
High National ADI	1.56	1.16	2.10	0.003
Low National ADI	1.06	1.01	1.12	0.022
Non-Hispanic White	1.08	1.02	1.16	0.015
Non-Hispanic Asian	1.12	0.77	1.63	0.558
Non-Hispanic Black	1.16	0.99	1.34	0.064
Hispanic	1.20	1.00	1.45	0.053
Non-Hispanic NHPI	1.91	1.10	3.29	0.021
Non-Hispanic AIAN	0.84	0.32	2.21	0.729
Non-Hispanic Multiracial	1.07	0.51	2.23	0.864
Has Been Homeless	1.29	1.11	1.49	0.001
Has Never Been Homeless	1.06	1.01	1.12	0.032
**4 day**				
Overall	1.09	1.04	1.14	<0.001
High National ADI	1.66	1.27	2.18	<0.001
Low National ADI	1.08	1.03	1.13	0.002
Non-Hispanic White	1.09	1.03	1.16	0.004
Non-Hispanic Asian	1.11	0.79	1.57	0.546
Non-Hispanic Black	1.13	0.99	1.30	0.080
Hispanic	1.26	1.07	1.50	0.006
Non-Hispanic NHPI	2.06	1.23	3.46	0.006
Non-Hispanic AIAN	0.62	0.24	1.61	0.328
Non-Hispanic Multiracial	0.94	0.47	1.88	0.857
Has Been Homeless	1.19	1.04	1.36	0.013

© 2025 Shannon EM et al. *JAMA Network Open.*

Has Never Been Homeless	1.08	1.03	1.14	0.002

© 2025 Shannon EM et al. *JAMA Network Open.*

eTable 4. Odds Ratios for Association of Extreme Heat Events and Mortality Based on Conditional Logistic Regression Models at Temperature Thresholds Defined Using National Center for Environmental Information Data From Zip Codes With Complete Temperature Data. Includes associations for single-day EHEs occurring on the same day of mortality through 4-day EHEs lasting from 3 days prior to a patient's death to the same day as the mortality event.

	90th percentile				***95th percentile***				***97.5th percentile***			
	**OR**	**95% CI**		**p-value**	**OR**	**95% CI**		**p-value**	**OR**	**95% CI**		**p-value**
Same-day	1.11	1.04	1.24	0.004	1.20	1.07	1.34	0.001	1.20	1.03	1.40	0.02
2 day	1.10	1.02	1.18	0.009	1.20	1.10	1.31	<0.001	1.22	1.08	1.38	0.002
3 day	1.15	1.07	1.22	<0.001	1.22	1.12	1.32	<0.001	1.22	1.09	1.36	<0.001
4 day	1.11	1.05	1.18	0.001	1.19	1.11	1.28	<0.001	1.19	1.08	1.31	0.001

© 2025 Shannon EM et al. *JAMA Network Open.*

eTable 5. Odds Ratios for Association of Extreme Heat Events and Mortality for Race, Ethnicity, Area Deprivation Index and Homeless Status Based on Conditional Logistic Regression Models at Temperature Thresholds Defined Using National Center for Environmental Information Data From Zip Codes With Complete Temperature Data. Includes associations for single-day EHEs occurring on the same day of mortality through 4-day EHEs lasting from 3 days prior to a patient's death to the same day as the mortality event. P-for-interaction for all assessments of effective modification >0.05 and not shown. High National ADI ≥75. Abbreviations. ADI, area deprivation index; AIAN, American Indian Alaska Native; CI, confidence interval; OR, odds ratio

**90th percentile**				
**Same-day**				
	**OR**	**95% CI**		**p-value**
Overall	1.13	1.04	1.24	0.004
High National ADI	1.54	1.10	2.15	0.011
Low National ADI	1.11	1.02	1.21	0.02
Non-Hispanic White	1.23	1.10	1.37	<0.001
Non-Hispanic Asian	0.95	0.55	1.66	0.867
Non-Hispanic Black	0.98	0.78	1.23	0.848
Hispanic	1.00	0.74	1.34	0.984
Non-Hispanic AIAN	1.76	0.55	5.63	0.341
Non-Hispanic Multiracial	0.45	0.13	1.58	0.213
Female	1.10	0.63	1.93	0.737
Male	1.14	1.04	1.24	0.004
Has Been Homeless	1.23	1.00	1.51	0.053
Has Never Been Homeless	1.12	1.02	1.23	0.022
**2 day**				
Overall	1.10	1.02	1.18	0.009
High National ADI	1.28	0.96	1.71	0.089
Low National ADI	1.09	1.01	1.17	0.024
Non-Hispanic White	1.17	1.07	1.28	0.001
Non-Hispanic Asian	1.05	0.67	1.64	0.842
Non-Hispanic Black	1.03	0.85	1.25	0.786
Hispanic	1.06	0.83	1.34	0.657
Non-Hispanic AIAN	0.95	0.33	2.68	0.916
Non-Hispanic Multiracial	0.65	0.26	1.67	0.373
Female	0.85	0.51	1.41	0.523
Male	1.11	1.03	1.19	0.006
Has Been Homeless	1.24	1.04	1.48	0.019
Has Never Been Homeless	1.08	0.99	1.17	0.07
**3 day**				
Overall	1.15	1.07	1.22	<0.001
High National ADI	1.54	1.20	1.97	0.001
Low National ADI	1.12	1.05	1.20	0.001
Non-Hispanic White	1.19	1.09	1.29	<0.001
Non-Hispanic Asian	1.07	0.73	1.57	0.738
Non-Hispanic Black	1.09	0.92	1.29	0.309
Hispanic	1.16	0.94	1.44	0.164
Non-Hispanic AIAN	1.36	0.59	3.11	0.469
Non-Hispanic Multiracial	0.90	0.41	2.00	0.796
Female	1.02	0.67	1.55	0.94
Male	1.15	1.08	1.23	<0.001

© 2025 Shannon EM et al. *JAMA Network Open.*

Has Been Homeless	1.30	1.11	1.52	0.001
Has Never Been Homeless	1.12	1.04	1.20	0.002
**4 day**				
Overall	1.11	1.05	1.18	0.001
High National ADI	1.45	1.14	1.83	0.002
Low National ADI	1.09	1.03	1.16	0.005
Non-Hispanic White	1.16	1.07	1.25	<0.001
Non-Hispanic Asian	1.15	0.81	1.64	0.432
Non-Hispanic Black	1.00	0.86	1.18	0.968
Hispanic	1.15	0.94	1.40	0.174
Non-Hispanic AIAN	1.17	0.53	2.58	0.69
Non-Hispanic Multiracial	0.71	0.32	1.57	0.397
Female	0.99	0.66	1.48	0.945
Male	1.12	1.05	1.19	<0.001
Has Been Homeless	1.17	1.00	1.35	0.044
Has Never Been Homeless	1.10	1.03	1.18	0.004
**95th percentile**				
**Same-day**				
Overall	1.20	1.07	1.34	0.001
High National ADI	1.67	1.06	2.62	0.028
Low National ADI	1.18	1.05	1.32	0.006
Non-Hispanic White	1.25	1.08	1.44	0.003
Non-Hispanic Asian	0.72	0.34	1.53	0.397
Non-Hispanic Black	1.06	0.80	1.41	0.695
Hispanic	1.30	0.90	1.89	0.167
Non-Hispanic AIAN	2.35	0.46	12.02	0.304
Non-Hispanic Multiracial	0.28	0.04	2.21	0.228
Female	1.43	0.66	3.08	0.362
Male	1.19	1.07	1.34	0.002
Has Been Homeless	1.44	1.11	1.88	0.006
Has Never Been Homeless	1.15	1.02	1.30	0.023
**2 day**				
Overall	1.20	1.10	1.31	<0.001
High National ADI	1.30	0.90	1.88	0.166
Low National ADI	1.19	1.09	1.31	<0.001
Non-Hispanic White	1.30	1.16	1.47	<0.001
Non-Hispanic Asian	0.72	0.38	1.35	0.3
Non-Hispanic Black	1.13	0.89	1.42	0.326
Hispanic	1.16	0.84	1.59	0.361
Non-Hispanic AIAN	1.03	0.26	4.05	0.972
Non-Hispanic Multiracial	0.44	0.09	2.03	0.29
Female	1.54	0.80	2.97	0.197
Male	1.19	1.09	1.31	<0.001
Has Been Homeless	1.31	1.05	1.64	0.018
Has Never Been Homeless	1.18	1.07	1.30	0.001
**3 day**				
Overall	1.22	1.12	1.32	<0.001
High National ADI	1.55	1.12	2.13	0.007
Low National ADI	1.20	1.10	1.30	<0.001
Non-Hispanic White	1.27	1.14	1.40	<0.001
Non-Hispanic Asian	0.93	0.56	1.54	0.763

© 2025 Shannon EM et al. *JAMA Network Open.*

Non-Hispanic Black	1.19	0.98	1.46	0.087
Hispanic	1.22	0.92	1.60	0.171
Non-Hispanic AIAN	1.22	0.41	3.63	0.716
Non-Hispanic Multiracial	0.73	0.24	2.30	0.595
Female	1.44	0.83	2.50	0.2
Male	1.21	1.12	1.31	<0.001
Has Been Homeless	1.39	1.14	1.68	0.001
Has Never Been Homeless	1.19	1.09	1.29	<0.001
**4 day**				
Overall	1.19	1.11	1.28	<0.001
High National ADI	1.55	1.16	2.07	0.003
Low National ADI	1.17	1.09	1.26	<0.001
Non-Hispanic White	1.23	1.12	1.35	<0.001
Non-Hispanic Asian	1.06	0.68	1.63	0.809
Non-Hispanic Black	1.11	0.93	1.34	0.254
Hispanic	1.29	1.01	1.65	0.041
Non-Hispanic AIAN	1.02	0.35	2.98	0.978
Non-Hispanic Multiracial	0.58	0.19	1.75	0.331
Female	1.26	0.76	2.09	0.372
Male	1.19	1.10	1.28	<0.001
Has Been Homeless	1.31	1.10	1.57	0.003
Has Never Been Homeless	1.17	1.08	1.26	<0.001
**97.5th percentile**				
**Same-day**				
Overall	1.20	1.03	1.40	0.022
High National ADI	1.56	0.78	3.09	0.207
Low National ADI	1.18	1.01	1.39	0.04
Non-Hispanic White	1.26	1.02	1.55	0.029
Non-Hispanic Asian	0.72	0.24	2.21	0.571
Non-Hispanic Black	0.91	0.61	1.35	0.624
Hispanic	1.44	0.87	2.39	0.156
Non-Hispanic AIAN	5.28	0.47	58.72	0.176
Non-Hispanic Multiracial	0.00	0.00	Inf	0.997
Female	1.38	0.53	3.58	0.512
Male	1.20	1.02	1.40	0.027
Has Been Homeless	1.56	1.10	2.20	0.013
Has Never Been Homeless	1.13	0.95	1.34	0.183
**2 day**				
Overall	1.22	1.08	1.38	0.002
High National ADI	1.42	0.83	2.43	0.201
Low National ADI	1.21	1.06	1.38	0.004
Non-Hispanic White	1.30	1.11	1.54	0.001
Non-Hispanic Asian	0.89	0.39	2.02	0.774
Non-Hispanic Black	1.07	0.78	1.46	0.679
Hispanic	1.30	0.85	2.00	0.231
Non-Hispanic AIAN	1.04	0.20	5.40	0.966
Non-Hispanic Multiracial	0.00	0.00	Inf	0.998
Female	1.73	0.74	4.06	0.206
Male	1.21	1.07	1.38	0.003
Has Been Homeless	1.28	0.96	1.72	0.098
Has Never Been Homeless	1.21	1.05	1.39	0.008

© 2025 Shannon EM et al. *JAMA Network Open.*

**3 day**				
Overall	1.22	1.09	1.36	<0.001
High National ADI	1.74	1.10	2.77	0.019
Low National ADI	1.19	1.07	1.33	0.002
Non-Hispanic White	1.24	1.07	1.43	0.004
Non-Hispanic Asian	0.91	0.46	1.79	0.781
Non-Hispanic Black	1.26	0.96	1.65	0.091
Hispanic	1.30	0.89	1.90	0.169
Non-Hispanic AIAN	1.45	0.42	5.00	0.554
Non-Hispanic Multiracial	0.38	0.05	3.15	0.366
Female	1.32	0.62	2.80	0.467
Male	1.22	1.09	1.36	0.001
Has Been Homeless	1.28	0.99	1.66	0.061
Has Never Been Homeless	1.20	1.07	1.36	0.002
**4 day**				
Overall	1.19	1.08	1.31	0.001
High National ADI	1.59	1.05	2.42	0.03
Low National ADI	1.17	1.06	1.29	0.003
Non-Hispanic White	1.20	1.06	1.37	0.006
Non-Hispanic Asian	0.92	0.51	1.64	0.767
Non-Hispanic Black	1.17	0.91	1.49	0.214
Hispanic	1.36	0.97	1.90	0.07
Non-Hispanic AIAN	1.45	0.42	5.00	0.554
Non-Hispanic Multiracial	0.38	0.05	3.15	0.366
Female	1.71	0.90	3.25	0.102
Male	1.18	1.07	1.30	0.001
Has Been Homeless	1.29	1.01	1.63	0.038
Has Never Been Homeless	1.17	1.05	1.30	0.005

© 2025 Shannon EM et al. *JAMA Network Open.*

Table 6. Odds Ratio for Association of Extreme Heat Events and Mortality for Gagne Score, Care Assessment Needs (CAN) Score and Cardiometabolic Condition Based on Conditional Logistic Regression Models at Temperature Thresholds Defined Using National Center for Environmental Information Data. Includes associations for single-day EHEs occurring on the same day of mortality through 4-day EHEs lasting from 3 days prior to a patient's death to the same day as the mortality event. P-for-interaction for all assessments of effective modification >0.05 and not shown. High National ADI ≥75; High Gagne>= 5; High CAN ≥90. Abbreviations: CI, confidence interval; OR, odds ratio

90th percentile	OR	95% CI		p-value
**Same-day**				
Overall	1.05	0.99	1.12	0.089
High Gagne	0.99	0.69	1.40	0.938
Low Gagne	1.05	0.99	1.12	0.082
High CAN	1.10	1.02	1.18	0.009
Low CAN	0.96	0.87	1.07	0.446
Chronic Kidney Disease	1.07	0.97	1.19	0.194
No Chronic Kidney Disease	1.04	0.97	1.12	0.24
Diabetes Mellitus	1.02	0.94	1.12	0.593
No Diabetes Mellitus	1.08	1.00	1.17	0.068
Heart Failure	1.03	0.92	1.14	0.657
No Heart Failure	1.06	0.99	1.14	0.082
Hypertension	1.05	0.98	1.12	0.144
No Hypertension	1.08	0.92	1.27	0.348
Ischemic Heart Disease	1.10	1.00	1.22	0.052
No Ischemic Heart Disease	1.03	0.95	1.10	0.486
Peripheral Vascular Disease	1.00	0.72	1.39	0.989
No Peripheral Vascular Disease	1.05	0.99	1.12	0.085
Stroke	1.09	0.96	1.25	0.199
No Stroke	1.04	0.98	1.11	0.205
**2 day**				
Overall	1.10	1.05	1.16	<0.001
High Gagne	0.93	0.68	1.27	0.652
Low Gagne	1.10	1.05	1.16	<0.001
High CAN	1.14	1.07	1.21	<0.001
Low CAN	1.03	0.94	1.12	0.592
Chronic Kidney Disease	1.13	1.03	1.24	0.007
No Chronic Kidney Disease	1.08	1.02	1.15	0.009
Diabetes Mellitus	1.07	0.99	1.15	0.092
No Diabetes Mellitus	1.13	1.05	1.21	0.001
Heart Failure	1.11	1.01	1.22	0.025
No Heart Failure	1.09	1.03	1.16	0.004
Hypertension	1.11	1.05	1.17	<0.001
No Hypertension	1.06	0.92	1.22	0.44

© 2025 Shannon EM et al. *JAMA Network Open.*

Ischemic Heart Disease	1.16	1.06	1.26	0.001
No Ischemic Heart Disease	1.07	1.00	1.14	0.04
Peripheral Vascular Disease	0.96	0.73	1.27	0.782
No Peripheral Vascular Disease	1.10	1.05	1.16	<0.001
Stroke	1.11	0.99	1.25	0.075
No Stroke	1.10	1.04	1.16	0.001
**3 day**				
Overall	1.10	1.05	1.15	<0.001
High Gagne	1.03	0.78	1.36	0.864
Low Gagne	1.10	1.05	1.15	<0.001
High CAN	1.13	1.07	1.20	<0.001
Low CAN	1.03	0.95	1.12	0.513
Chronic Kidney Disease	1.15	1.06	1.25	0.001
No Chronic Kidney Disease	1.08	1.02	1.14	0.011
Diabetes Mellitus	1.07	1.00	1.15	0.046
No Diabetes Mellitus	1.12	1.05	1.20	<0.001
Heart Failure	1.12	1.02	1.22	0.013
No Heart Failure	1.09	1.03	1.15	0.002
Hypertension	1.11	1.06	1.17	<0.001
No Hypertension	1.00	0.88	1.14	0.993
Ischemic Heart Disease	1.13	1.05	1.23	0.002
No Ischemic Heart Disease	1.08	1.02	1.15	0.009
Peripheral Vascular Disease	1.00	0.77	1.29	0.982
No Peripheral Vascular Disease	1.10	1.05	1.16	<0.001
Stroke	1.09	0.98	1.21	0.113
No Stroke	1.10	1.04	1.16	<0.001
**4 day**				
Overall	1.07	1.02	1.12	0.005
High Gagne	1.01	0.77	1.32	0.967
Low Gagne	1.07	1.02	1.12	0.004
High CAN	1.10	1.04	1.16	0.001
Low CAN	1.00	0.92	1.08	0.975
Chronic Kidney Disease	1.10	1.01	1.19	0.021
No Chronic Kidney Disease	1.05	1.00	1.11	0.064
Diabetes Mellitus	1.04	0.98	1.11	0.223
No Diabetes Mellitus	1.09	1.02	1.16	0.006
Heart Failure	1.11	1.02	1.20	0.016
No Heart Failure	1.05	1.00	1.11	0.069
Hypertension	1.08	1.03	1.14	0.001
No Hypertension	0.96	0.84	1.09	0.504
Ischemic Heart Disease	1.09	1.01	1.17	0.029

© 2025 Shannon EM et al. *JAMA Network Open.*

No Ischemic Heart Disease	1.06	1.00	1.12	0.057
Peripheral Vascular Disease	0.93	0.72	1.19	0.553
No Peripheral Vascular Disease	1.07	1.02	1.12	0.003
Stroke	1.12	1.01	1.24	0.03
No Stroke	1.05	1.00	1.11	0.037
**95th percentile same-day**				
Overall	1.11	1.03	1.20	0.005
High Gagne	1.17	0.74	1.84	0.499
Low Gagne	1.11	1.03	1.20	0.006
High CAN	1.16	1.06	1.27	0.001
Low CAN	1.02	0.90	1.16	0.773
Chronic Kidney Disease	1.09	0.96	1.25	0.196
No Chronic Kidney Disease	1.12	1.03	1.23	0.011
Diabetes Mellitus	1.06	0.95	1.19	0.273
No Diabetes Mellitus	1.16	1.05	1.28	0.004
Heart Failure	1.18	1.03	1.35	0.016
No Heart Failure	1.09	1.00	1.18	0.066
Hypertension	1.10	1.02	1.19	0.016
No Hypertension	1.19	0.97	1.46	0.094
Ischemic Heart Disease	1.19	1.05	1.34	0.008
No Ischemic Heart Disease	1.08	0.98	1.18	0.11
Peripheral Vascular Disease	0.89	0.57	1.37	0.588
No Peripheral Vascular Disease	1.12	1.04	1.21	0.003
Stroke	1.25	1.06	1.47	0.009
No Stroke	1.08	1.00	1.18	0.058
**2 day**				
Overall	1.10	1.04	1.17	0.002
High Gagne	1.01	0.68	1.49	0.979
Low Gagne	1.11	1.04	1.18	0.002
High CAN	1.16	1.07	1.25	<0.001
Low CAN	1.00	0.89	1.11	0.933
Chronic Kidney Disease	1.12	1.00	1.25	0.047
No Chronic Kidney Disease	1.10	1.02	1.18	0.016
Diabetes Mellitus	1.05	0.96	1.16	0.259
No Diabetes Mellitus	1.15	1.05	1.24	0.002
Heart Failure	1.21	1.08	1.36	0.001
No Heart Failure	1.06	0.98	1.14	0.127
Hypertension	1.11	1.04	1.18	0.003
No Hypertension	1.07	0.90	1.28	0.425
Ischemic Heart Disease	1.20	1.08	1.33	0.001

© 2025 Shannon EM et al. *JAMA Network Open.*

No Ischemic Heart Disease	1.06	0.98	1.14	0.157
Peripheral Vascular Disease	0.81	0.56	1.17	0.261
No Peripheral Vascular Disease	1.11	1.05	1.19	0.001
Stroke	1.18	1.02	1.36	0.022
No Stroke	1.09	1.01	1.16	0.02
**3 day**				
Overall	1.14	1.08	1.20	<0.001
High Gagne	1.20	0.86	1.68	0.287
Low Gagne	1.14	1.07	1.20	<0.001
High CAN	1.18	1.10	1.26	<0.001
Low CAN	1.05	0.95	1.16	0.314
Chronic Kidney Disease	1.20	1.09	1.32	<0.001
No Chronic Kidney Disease	1.11	1.04	1.19	0.002
Diabetes Mellitus	1.09	1.01	1.19	0.034
No Diabetes Mellitus	1.18	1.09	1.27	<0.001
Heart Failure	1.20	1.09	1.33	<0.001
No Heart Failure	1.11	1.04	1.19	0.002
Hypertension	1.14	1.08	1.21	<0.001
No Hypertension	1.10	0.94	1.29	0.227
Ischemic Heart Disease	1.18	1.08	1.30	0.001
No Ischemic Heart Disease	1.11	1.04	1.19	0.002
Peripheral Vascular Disease	0.89	0.65	1.23	0.488
No Peripheral Vascular Disease	1.15	1.08	1.21	<0.001
Stroke	1.20	1.06	1.36	0.004
No Stroke	1.12	1.05	1.19	<0.001
**4 day**				
Overall	1.11	1.05	1.17	<0.001
High Gagne	1.09	0.80	1.50	0.585
Low Gagne	1.11	1.05	1.17	<0.001
High CAN	1.16	1.09	1.23	<0.001
Low CAN	1.01	0.92	1.11	0.827
Chronic Kidney Disease	1.16	1.06	1.27	0.002
No Chronic Kidney Disease	1.09	1.02	1.16	0.01
Diabetes Mellitus	1.08	1.00	1.16	0.058
No Diabetes Mellitus	1.14	1.06	1.22	<0.001
Heart Failure	1.16	1.06	1.28	0.002
No Heart Failure	1.09	1.02	1.16	0.008
Hypertension	1.12	1.06	1.18	<0.001
No Hypertension	1.05	0.91	1.21	0.511
Ischemic Heart Disease	1.12	1.03	1.23	0.009

© 2025 Shannon EM et al. *JAMA Network Open.*

No Ischemic Heart Disease	1.10	1.03	1.17	0.003
Peripheral Vascular Disease	0.95	0.71	1.27	0.722
No Peripheral Vascular Disease	1.11	1.06	1.17	<0.001
Stroke	1.18	1.05	1.32	0.006
No Stroke	1.09	1.03	1.16	0.003
**97.5th percentile same-day**				
Overall	1.18	1.07	1.30	0.001
High Gagne	1.38	0.80	2.38	0.243
Low Gagne	1.17	1.06	1.29	0.001
High CAN	1.25	1.12	1.40	<0.001
Low CAN	1.03	0.87	1.23	0.71
Chronic Kidney Disease	1.18	1.00	1.41	0.053
No Chronic Kidney Disease	1.18	1.05	1.32	0.006
Diabetes Mellitus	1.14	0.99	1.31	0.076
No Diabetes Mellitus	1.21	1.07	1.38	0.003
Heart Failure	1.34	1.13	1.60	0.001
No Heart Failure	1.12	1.00	1.25	0.053
Hypertension	1.17	1.06	1.30	0.002
No Hypertension	1.22	0.94	1.57	0.134
Ischemic Heart Disease	1.28	1.09	1.50	0.003
No Ischemic Heart Disease	1.13	1.00	1.27	0.044
Peripheral Vascular Disease	0.90	0.49	1.65	0.736
No Peripheral Vascular Disease	1.19	1.08	1.31	0.001
Stroke	1.30	1.05	1.61	0.018
No Stroke	1.15	1.04	1.28	0.009
**2 day**				
Overall	1.18	1.10	1.28	<0.001
High Gagne	1.30	0.82	2.07	0.267
Low Gagne	1.18	1.09	1.28	<0.001
High CAN	1.24	1.13	1.37	<0.001
Low CAN	1.06	0.92	1.23	0.397
Chronic Kidney Disease	1.20	1.05	1.38	0.01
No Chronic Kidney Disease	1.18	1.07	1.29	0.001
Diabetes Mellitus	1.13	1.00	1.27	0.047
No Diabetes Mellitus	1.23	1.11	1.37	<0.001
Heart Failure	1.37	1.18	1.57	<0.001
No Heart Failure	1.11	1.02	1.22	0.024
Hypertension	1.20	1.10	1.30	<0.001
No Hypertension	1.12	0.90	1.38	0.308
Ischemic Heart Disease	1.29	1.13	1.47	<0.001

© 2025 Shannon EM et al. *JAMA Network Open.*

No Ischemic Heart Disease	1.13	1.03	1.25	0.012
Peripheral Vascular Disease	0.85	0.52	1.40	0.528
No Peripheral Vascular Disease	1.19	1.10	1.29	<0.001
Stroke	1.22	1.02	1.46	0.028
No Stroke	1.18	1.08	1.28	<0.001
**3 day**				
Overall	1.19	1.11	1.27	<0.001
High Gagne	1.29	0.86	1.94	0.215
Low Gagne	1.18	1.10	1.27	<0.001
High CAN	1.22	1.13	1.33	<0.001
Low CAN	1.11	0.98	1.25	0.11
Chronic Kidney Disease	1.24	1.09	1.40	0.001
No Chronic Kidney Disease	1.16	1.07	1.26	<0.001
Diabetes Mellitus	1.14	1.03	1.26	0.011
No Diabetes Mellitus	1.22	1.12	1.34	<0.001
Heart Failure	1.30	1.15	1.47	<0.001
No Heart Failure	1.14	1.05	1.24	0.002
Hypertension	1.21	1.12	1.30	<0.001
No Hypertension	1.05	0.87	1.27	0.583
Ischemic Heart Disease	1.26	1.12	1.41	<0.001
No Ischemic Heart Disease	1.15	1.05	1.25	0.001
Peripheral Vascular Disease	0.95	0.64	1.43	0.822
No Peripheral Vascular Disease	1.19	1.11	1.28	<0.001
Stroke	1.25	1.07	1.46	0.005
No Stroke	1.17	1.08	1.26	<0.001
**4 day**				
Overall	1.17	1.10	1.24	<0.001
High Gagne	1.19	0.82	1.73	0.371
Low Gagne	1.17	1.10	1.24	<0.001
High CAN	1.24	1.15	1.33	<0.001
Low CAN	1.03	0.92	1.15	0.582
Chronic Kidney Disease	1.23	1.10	1.37	<0.001
No Chronic Kidney Disease	1.14	1.06	1.23	0.001
Diabetes Mellitus	1.13	1.03	1.24	0.008
No Diabetes Mellitus	1.20	1.10	1.30	<0.001
Heart Failure	1.23	1.10	1.38	<0.001
No Heart Failure	1.14	1.06	1.23	<0.001
Hypertension	1.18	1.11	1.27	<0.001
No Hypertension	1.07	0.90	1.27	0.448
Ischemic Heart Disease	1.18	1.06	1.31	0.002

© 2025 Shannon EM et al. *JAMA Network Open.*

No Ischemic Heart Disease	1.16	1.07	1.25	<0.001
Peripheral Vascular Disease	0.92	0.63	1.34	0.672
No Peripheral Vascular Disease	1.18	1.10	1.25	<0.001
Stroke	1.24	1.08	1.43	0.003
No Stroke	1.15	1.07	1.23	<0.001

© 2025 Shannon EM et al. *JAMA Network Open.*
