# Supplementary material for: Extreme Heat, Social Factors, and Mortality Among California Veterans With Cardiometabolic Disease
Source: JAMA Netw Open. 2025 Nov 25;8(11):e2545524. doi: 10.1001/jamanetworkopen.2025.45524 (PMC12648351; doi:10.1001/jamanetworkopen.2025.45524)
Supplement: Supplement 2. — Data Sharing Statement [file jamanetwopen-e2545524-s002.pdf]

Data Sharing Statement

Shannon. Extreme Heat, Social Factors, and Mortality Among California Veterans With Cardiometabolic Disease. *JAMA Netw Open*. Published November 25, 2025. doi:10.1001/jamanetworkopen.2025.45524

Data
Data available: No

Additional Information
Explanation for why data not available: Data from the Veterans Health Administration is proprietary and is not able to be shared.
